# First‐in‐Human Phase 1 Study to Evaluate the Clinical Pharmacology Properties of RBN‐3143, a Novel Inhibitor of Mono‐Adenosine Diphosphate Ribosyltransferase‐PARP14

**DOI:** 10.1002/cpdd.1539

**Published:** 2025-04-30

**Authors:** Thomas M. Polasek, Alexandra Cole, Viviana Bozón, Erika Manyak, Jonathan Novak, Barbara Yang, Briohny A. Johnston, Sudha Parasuraman, Kushal J. Paneliya, Virna Schuck

**Affiliations:** ^1^ CMAX Clinical Research Pty Ltd Adelaide South Australia Australia; ^2^ Centre for Medicines Use and Safety Monash University Melbourne Victoria Australia; ^3^ New Zealand Clinical Research Auckland New Zealand; ^4^ Ribon Therapeutics Inc. Cambridge MA USA

**Keywords:** first‐in‐human study, PARP14, pharmacokinetics, RBN‐3143, safety, tolerability

## Abstract

RBN‐3143 is an inhibitor of PARP14 in development for inflammatory diseases. Multiple assessments were conducted to evaluate the clinical pharmacology properties of RBN‐3134. A randomized, double‐blind, placebo‐controlled study assigned healthy volunteers (HVs) to single ascending doses (SADs) (25‐1000 mg) or multiple ascending doses (MADs) (150, 300, and 500 mg twice daily [BID] for 14 days) of RBN‐3143 or placebo. An open‐label, randomized, 3‐period, cross‐over study evaluated the effects of food and pantoprazole (40 mg once daily [QD]) on the pharmacokinetics of RBN‐3143 (500 mg), and a pharmacokinetic drug–drug interaction study with oral midazolam (2 mg) determined whether RBN‐3143 (300 mg BID for 14 days) is an inducer of cytochrome P4503A4 (CYP3A4). The most common treatment‐related treatment‐emergent adverse events in subjects taking RBN‐3143 were headache, nausea, vomiting, and elevated serum creatinine. In the SAD, RBN‐3143 C_max_ and AUC_inf_ increased with dose, and T_max_ was 2 hours. RBN‐3143 was cleared from plasma with an apparent terminal half‐life ranging from 3 to 11 hours. In the MAD, C_max_ and AUC_inf_ increased 1.5‐ and 1.6‐fold, respectively, following 14 days of 150, 300, and 500 mg BID dosing. Dosing of RBN‐3143 with food resulted in higher C_max_ and AUC_inf_ ratios of 1.74 and 1.42, respectively. Coadministration with pantoprazole did not impact RBN‐3143 exposure. RBN‐3143 was an inducer of CYP3A4 in most but not all subjects, with mean midazolam C_max_ and AUC_inf_ ratios of 0.92 and 0.88, respectively. The clinical pharmacology properties of RBN‐3143 in HVs support further development for inflammatory diseases.

The PARP proteins are a family of 17 enzymes that regulate fundamental cellular processes, including gene expression, protein degradation, and multiple cellular stress responses.^1^, ^2^ PARPs covalently modify target proteins with ADP‐ribose, a process called ADP‐ribosylation. Mono‐adenosine diphosphate (ADP) ribosyltransferase (mono‐ART)‐PARP14 (PARP14) is 1 of 3 interferon‐inducible, macrodomain‐containing PARPs that promote signaling by Type 2 helper T cell (TH2) and Type 17 helper T cell (TH17) cytokines by acting as a coactivator of STAT6‐ and STAT3‐driven transcription.^3^, ^4^ PARP14 is upregulated in tissues with inflammatory disease, such as skin lesions from atopic dermatitis and psoriasis patients,^5^ and in endobronchial biopsies from patients with asthma.^6^ Genetic deletion or catalytic inhibition of PARP14 has been shown to block interleukin (IL)‐4/STAT6 signaling in macrophages in vitro^7^, ^8^ and to suppress pathogenic changes associated with allergic airway disease in mouse models.^9^, ^10^ Antibodies and small molecules suppressing TH2/TH17‐cytokine signaling and alarmins are either approved or being investigated as treatments for multiple inflammatory diseases, such as atopic dermatitis, asthma, chronic rhinosinusitis, and eosinophilic esophagitis.^11^, ^12^, ^13^

Nonclinical studies showed that RBN‐3143 (Figure 1) is a nicotinamide adenine dinucleotide (NAD+)‐competitive inhibitor of PARP14 across mouse, rat, dog, and human species. In vivo, oral administration of RBN‐3143 caused the suppression of disease phenotypes in various rodent models of lung and skin inflammation. For example, in an Alternaria alternata–induced asthma model, RBN‐3143 significantly suppressed airway mucus, serum immunoglobulin E (IgE) levels, and the accumulation of immune cells, inflammatory cytokines, and alarmins in the lungs. Repeat‐dose toxicology studies in rats and dogs identified the thyroid gland, the adrenal glands, the liver, bone, skeletal muscle, secondary lymphoid tissues, and the male reproductive tract as potential target organs of toxicity (unpublished data).

**Figure 1:**
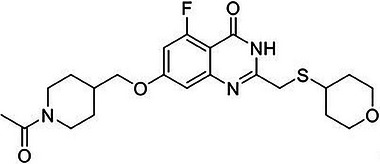
Molecular structure of RBN‐3143.

Nonclinical pharmacokinetic (PK) studies in mouse, rat, dog, and monkey showed favorable absorption, distribution, metabolism, and excretion (ADME) properties for RBN‐3143. In vitro data indicated a potential impact of acid‐reducing agents and food on the solubility and, consequently, absorption of RBN‐3143. RBN‐3143 appears to be predominantly metabolized by cytochrome P4503A4 (CYP3A4) and has the potential for clinically significant induction of CYP3A4 (unpublished data).

Given the upregulation of PARP14 in tissues with inflammatory diseases, the central role of PARP14 in TH2‐ and TH17‐driven cytokine signaling, and the common underlying biology of many inflammatory diseases, RBN‐3143 is being developed as an orally active inhibitor of PARP14. Therefore, the objective of this study was to evaluate the clinical pharmacology characteristics of RBN‐3143, including single‐dose and multiple‐dose PKs, susceptibility to a food effect (FE), and the potential for drug–drug interactions (DDIs) with proton pump inhibitors (PPIs) and drugs cleared predominantly by CYP3A4.

## Methods

### Study Design

The overall study design is shown with sequential cohorts in Figure S1. The single ascending dose (SAD) and multiple ascending dose (MAD) cohorts were double‐blind, randomized, placebo‐controlled, dose‐escalation studies to evaluate the safety, tolerability, and PK of RBN‐3143 following single and multiple doses. There were 7 SAD cohorts comprising 8 healthy volunteers (HVs) who received single oral doses of either RBN‐3143 (25, 50, 100, 150, 300, 600, and 1000 mg) or placebo (6 active, 2 placebo). There were 3 MAD cohorts comprising 8 HVs who received multiple oral doses of either RBN‐3143 (150, 300, and 500 mg) or placebo twice daily (BID) for 14 days (6 active, 2 placebo). Each cohort had 2 sentinel participants (1 active, 1 placebo). Safety and tolerability were determined prior to dosing of the remaining subjects in the cohort. Higher dose cohorts were started following the review of safety, tolerability, and PK (when available) data by a safety review committee (SRC).

The FE/PPI cohort was an open‐label, randomized, 3‐period, cross‐over study to evaluate the effects of a standardized high‐fat breakfast (30 minutes prior to RBN‐3143 dosing) and the PPI pantoprazole (40 mg once daily [QD] for 10 days with RBN‐3143 taken on day 7) on the safety, tolerability, and PK of RBN‐3143 following a single 500 mg oral dose. Thirteen HVs were randomized to either sequence A (fasted, fed, PPI) or sequence B (fed, fasted, PPI). Each period was separated by a 3‐day wash‐out period.

The DDI cohort was an open‐label, 2‐treatment period, fixed‐sequence study to evaluate the effects of RBN‐3143 on the PKs of midazolam, a CYP3A4 sensitive probe substrate. Twelve HVs were administered a single 2 mg oral dose of midazolam on day 1, followed by RBN‐3143 300 mg BID from day 3 to day 16 (14‐day treatment), with a second dose of 2 mg oral midazolam taken on day 16.

### Study Population

Healthy males and females aged 18‐65 years with body mass indexes of ≥18.0 and ≤35.0 kg/m^2^, respectively, were eligible for the study. Subjects had to refrain from tobacco smoking, caffeine, alcohol, xanthine‐containing foods, and grapefruit juice prior to and during the study. Prescription and herbal medications were also prohibited. Women of childbearing potential had to be taking 2 highly effective forms of contraception. Table S1 lists the full inclusion and exclusion criteria for the study. All subjects provided written informed consent prior to screening.

### Study Assessments

Safety and tolerability were determined by assessing treatment‐emergent adverse events (TEAEs), vital signs, laboratory test results (eg, hematology, biochemistry, coagulation, thyroid function tests, and urinalysis), electrocardiograms, and physical examination findings (Table S2 shows the schedules of assessments). Adverse events were graded as per the current version of the common terminology criteria for adverse events (CTCAE) criteria and categorized as mild (grade 1), moderate (grade 2), severe (grade 3), life‐threatening (grade 4), death (grade 5) or serious (SAE) according to standard definitions.^14^, ^15^ Causal relationships of TEAEs to RBN‐3143 administration were judged by the investigators as either “not related” or “related” (treatment‐related TEAEs). All TEAEs were coded using the Medical Dictionary of Regulatory Activities (MedRDA V24.0). Randomized subjects who received at least 1 dose of RBN‐3143 or placebo were included in the safety population for analysis.

### Pharmacokinetic Sampling and Bioanalytical Methods

The PK of RBN‐3143 was determined by collecting serial blood samples and pooled urine samples according to the sampling schedules shown in Table S3. Liquid chromatography–tandem mass spectrometry was used to quantify the plasma and urine concentrations of RBN‐3143 and plasma concentrations of midazolam (DDI cohort only) according to the following validated methods.

RBN‐3143 and the internal standard (D4 analogue of RBN‐3143) were extracted from plasma samples (50 µL aliquots) by a protein precipitation procedure using Waters (Boston, MA, USA) Ostro protein precipitation plates, while aliquots of urine samples (50 µL) were prepared using a dilution method. Analytes were separated by HPLC on a Waters Atlantis T3 analytical column using a gradient mobile phase (mobile phase A: 10% acetonitrile, 90% water, 10 mM ammonium acetate, 0.3% formic acid; mobile phase B: 90% acetonitrile, 10% water, 10 mM ammonium acetate, 0.3% formic acid) run at 0.4 mL/min with initial mobile phase B concentration of 30%. The eluates were monitored by an API4000 MS/MS detector in positive multiple reaction monitoring (MRM) mode (m/z 450.1 to m/z 310.9 for RBN‐3143 and m/z 454.2 to mz/ 144.1 for internal standard). Calibration curves were linear from 10.0 to 10,000 ng/mL in plasma and from 1.0 to 1000 ng/mL in urine. The intra‐assay precision was between 4.0% and 8.0%, and accuracy was between −12.8% and 11.2%. The inter‐assay precision was between 6.0% and 12.0%, and accuracy was between 4.4% and 4.4%.

Plasma aliquots of 50 µL were analyzed for midazolam using a proprietary method developed by Agilex Biolabs (Adelaide, SA, Australia). The LC/MS/MS method used the D4 analogue of midazolam as the internal standard. Calibration curves were linear from 0.10 to 100 ng/mL. The intra‐assay precision was between 1.3% and 12.3%, and accuracy was between −5.1% and 3.9%. The inter‐assay precision was between 2.1% and 7.9%, and accuracy was between −3.5% and 1.9%.

### Data Analysis

Statistical analyses were conducted using SAS Version 9.4 (SAS Institute Inc., Cary, NC, USA). Pharmacokinetic analyses and the analysis of the food effect and DDIs were conducted using Phoenix WinNonlin Version 8.1 (Certara, Princeton, NJ, USA). Safety data and PK data were summarized by cohort using descriptive statistics.

In the calculation of the mean concentration profiles, concentrations of RBN‐3143 that were less than the lower limit of quantification were designated a value of zero. PK parameters were calculated for all subjects with no protocol violations and with sufficient data points on the concentration–time profile. Pharmacokinetic parameters included area under the concentration–time curve from time 0 to infinity (AUC_inf_), AUC from time 0 to the last measurable plasma concentration (AUC_last_), AUC over the dosing interval (AUC_tau_), maximum plasma concentration (C_max_), maximum plasma concentration at steady‐state (C_max,ss_), time to maximum plasma concentration (T_max_), time to maximum plasma concentration at steady‐state (T_max,ss_), and the apparent terminal half‐life (t_1/2_).

A linear mixed effects model was used to analyze the effects of food and pantoprazole on RBN‐3143 PKs and to analyze the RBN‐3143 impact on midazolam PKs. The model used the ln‐transformed PK parameters (C_max_ and AUC) as the dependent variable, dosing condition (fed vs fasted) or treatment (RBN‐3143 alone vs with pantoprazole, midazolam alone vs. with RBN‐3143) as fixed effects, and participant nested within sequence as a random effect. Point estimates and 90% confidence intervals to assess the effect of food and pantoprazole (PPI) on the PK were calculated as fed/fasted and +PPI/−PPI ratios for AUC and C_max_. Likewise, for the effect of RBN‐3143 on midazolam PK (MDZ_+RBN‐3143_/MDZ_−RBN‐3143_).

### Study Sites

The SAD, MAD, and PPI/FE parts of the study were conducted at CMAX Clinical Research in Adelaide, Australia, and the midazolam DDI part of the study was conducted at New Zealand Clinical Research in Auckland, New Zealand.

### Study Oversight

The study is registered at ClinicalTrials.gov (NCT05215808). It was conducted according to the Declaration of Helsinki, the ICH Guidelines for Good Clinical Practice, and all applicable regulatory requirements. Ethical oversight was provided by the Bellberry Human Research Ethics Committee in Australia and the Southern Health and Disability Ethics Committee in New Zealand. During the study, an SRC comprising the Principal Investigator, the Sponsor's chief medical officer, and an independent medical monitor, evaluated all data to minimize potential risks to study subjects.

## Results

### Subject Demographics and Baseline Characteristics

The subject demographics and baseline characteristics for all cohorts are shown in Table S4. Fifty‐three subjects were enrolled in the SAD cohorts, of whom 40 received RBN‐3143 and 13 received a placebo. Twenty‐four subjects were enrolled in the MAD cohorts, of whom 18 received RBN‐3143 and 6 received a placebo. The FE/PPI cohort enrolled 13 subjects, with 1 subject withdrawing after period 2 for personal reasons. The DDI cohort enrolled 15 subjects. One subject was discontinued from the study due to a treatment‐related TEAE, while 2 withdrew consent (see Safety and Tolerability in the FE/PPI and DDI Cohorts).

### Safety and Tolerability in the SAD and MAD Cohorts

All TEAEs were of grade 1 or 2 severity, and no serious TEAEs were reported. Tables S5 and S6 show related TEAEs by preferred term in the SAD and MAD cohorts, respectively. Apart from the cases of elevated TSH, and mildly elevated serum creatinine on day 7 (1.4592 mg/dL, ULN = 1.2443 mg/dL) and day 14 (1.4706 mg/dL) in 1 subject taking RBN‐3143 300 mg BID (n = 1, 5.6%) (Table S6), there were no other clinically significant treatment‐related changes in clinical laboratory parameters, vital signs, or ECG parameters.

### Safety and Tolerability in the FE/PPI and DDI Cohorts

Of the 13 subjects who received RBN‐3143 in the FE/PPI cohort, 3 experienced at least 1 treatment‐related TEAE, and all were mild in severity. These included nausea (n = 2, 15%), a dental abscess (n = 1, 8%), decreased appetite (n = 1, 8%), loose stool (n = 1, 8%), and tiredness (n = 1, 8%).

Of the 15 subjects who received 300 mg BID of RBN‐3143 in the DDI cohort, 11 experienced at least 1 treatment‐related TEAE. These included headache (n = 5, 33.3%), fatigue (n = 2, 13.3%), lower back pain (n = 2, 13.3%), constipation (n = 1, 6.6%), abdominal discomfort (n = 1, 6.6%), abdominal cramps (n = 1, 6.6%), limb pruritis (n = 1, 6.6%), myalgia (n = 1, 6.6%), second‐degree heart block (n = 1, 6.6%) and intermittent runs of wide‐complex extra systoles (n = 1, 6.6%) (all grade 1 severity). Four subjects had elevated serum creatinine above the upper limit of normal (UNL; 1.24 mg/dL) on day 9 of the study (range 1.29 to 1.44 mg/dL) (n = 4, 33%). One of these subjects discontinued dosing on day 12 as serum creatinine continued to increase (from 1.42 to 1.65 mg/dL, eGFR = 55 mL/min down from 93 mL/min at baseline; grade 1). Serum creatinine levels returned to baseline soon after study drug discontinuation. The subject was asymptomatic, with normal chemistry, blood pressure, and urine examination. Renal function was assessed by cystatin C‐based calculations (an alternate method) and was normal, indicating no change to renal function. Another subject had grade 2 transaminase elevation by day 5 of treatment with RBN‐3143. Maximum ALT concentration was 148 U/L (normal range 5‐40 U/L) and AST concentration was 68 U/L (normal range 10‐50 U/L). The subject was asymptomatic with normal bilirubin concentrations and other chemistries. These were judged to be clinically significant by the investigator and the subject was discontinued from the study simultaneously for personal reasons. Transaminase levels returned to baseline soon after the study drug was discontinued.

### Pharmacokinetics of RBN‐3143 in the SAD and MAD Cohorts

**Figure 2:**
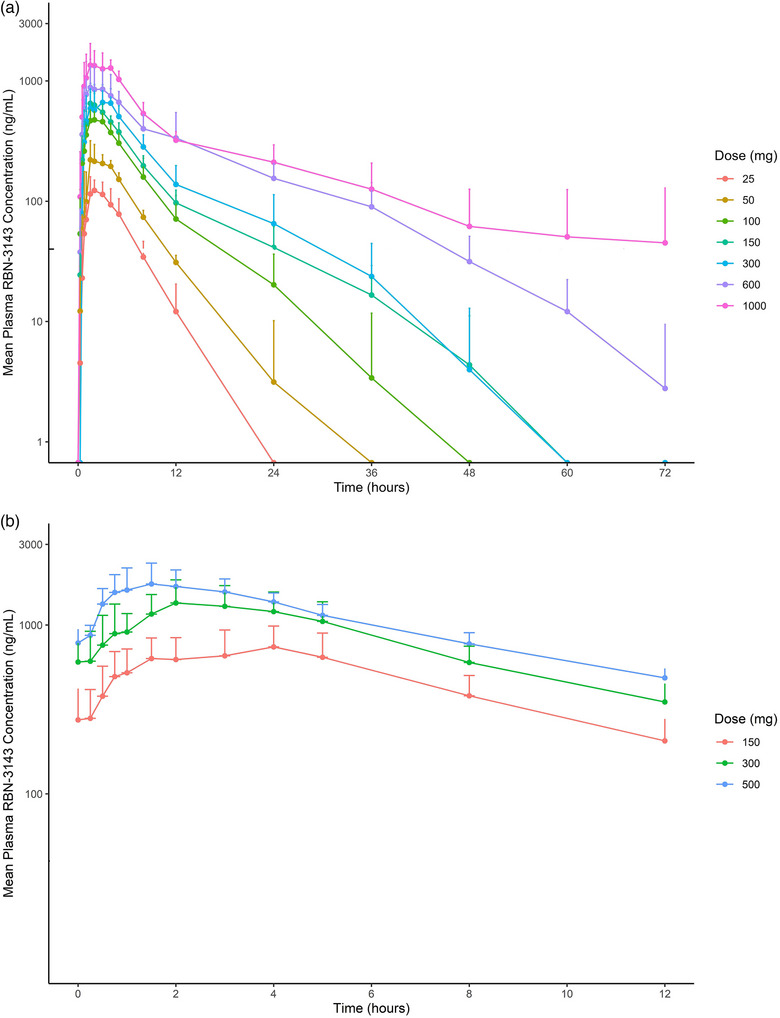
Plasma concentration–time profiles of RBN‐3143 after single doses (a) and after twice daily dosing for 14 days (b).

The PK parameters of RBN‐3143 after single and multiple doses are shown in Tables 1 and 2. RBN‐3143 was rapidly absorbed following oral administration, with peak concentrations achieved by approximately 2.0 hours (Figure 2a). After a single dose, plasma C_max_ and AUC_inf_ generally increase in a dose‐proportional manner from 25 to 150 mg and then from 300 to 1000 mg. Plasma t_1/2_ ranged from 2.9 to 10.9 hours. The PK of RBN‐3143 on day 1 of the MAD cohorts was consistent with the data observed in the SAD cohort at the same dose level. At steady‐state, RBN‐3143 plasma AUC increased approximately 1.6‐fold compared with day 1 following 14 days of 150, 300, and 500 mg BID dosing (Figure 2b), indicating that significant autoinduction of metabolic clearance via CYP3A4 was unlikely. Similarly, accumulations of approximately 1.5‐fold were observed for C_max_. Approximately 16% of the RBN‐3143 dose was excreted in urine unchanged.

**Table 1:** Pharmacokinetics of RN‐3143 After Single Ascending Doses

Dose level	Dose (mg)	Statistic	T_max_ ^a^ (hour)	C_max_ (ng/mL)	AUC_inf_ (h•ng/mL)	t_1/2_ (hour)
1	25	Mean (SD) Geometric mean (Geo CV%)	2.00 [1.10‐3.00]	138 (38.4) 134 (29.5%)	752 (186) 734 (24.7%)	2.88 (0.17) 2.88 (6.0%)
2	50	Mean (SD) Geometric mean (Geo CV%)	2.00 [1.25‐4.00]	269 (90.7) 259 (30.4%)	1560 (249) 1540 (16.4%)	3.50 (1.03) 3.4 (26.4%)
3	100	Mean (SD) Geometric mean (Geo CV%)	1.75 [0.75‐5.00]	556 (129) 544 (22.4%)	3610 (816) 3540 (22.9%)	5.31 (2.15) 4.94 (44.5%)
4	150	Mean (SD) Geometric mean (Geo CV%)	2.00 [1.50‐4.00]	668 (147) 653 (24.5%)	5050 (851) 5000 (16.8%)	8.58 (2.05) 8.30 (31.2%)
5	300	Mean (SD) Geometric mean (Geo CV%)	2.00 [1.5‐4.00]	771 (225) 747 (28.4%)	6440 (1800) 6230 (29.5%)	7.61 (2.95 4) 7.13 (43.6%)
6	600	Mean (SD) Geometric mean (Geo CV%)	1.75 [1.00‐12.0]	1240 (356) 1204 (30.5%)	11,900 (2730) 11,722 (23.2%)	13.78 (12.87) 10.92 (73.6%)
6a	1000	Mean (SD) Geometric mean (Geo CV%)	2.02 [1.50‐4.00]	1500 (405) 1460 (27.3%)	17,700 (7320) 16,500 (44.8%)	10.61 (4.32) 9.88 (43.2%)

One subject in cohort 2 was excluded from the summary due to vomiting.

a T_max_ data presented as median [min‐max].

**Table 2:** Pharmacokinetics of RN‐3143 After Multiple Ascending Doses

Dose level	Dose (mg)	Statistic	T_max_ ^a^ (hour)	C_max_ (ng/mL)	AUC_12_ (h•ng/mL)	t_1/2_ (hour)
7 Day 1	150	Mean (SD) Geometric mean (Geo CV%)	2.50 [0.75‐3.98]	623 (192) 597 (33.3%)	3610 (1180) 3470 (30.3%)	4.52 (2.48) 4.13 (45.2%)
7 Day 14	150	Mean (SD) Geometric mean (Geo CV%)	2.50 [0.75‐4.00]	861 (791) 846 (20.6%)	5660 (1170) 5570 (20.2%)	NC
8 Day 1	300	Mean (SD) Geometric mean (Geo CV%)	2.50 [1.50‐4.02]	925 (196) 907 (21.7%)	5590 (846) 5540 (15.8%)	4.19 (0.69) 4.15 (16.3%)
8 Day 14	300	Mean (SD) Geometric mean (Geo CV%)	2.00 [1.50‐4.00]	1470 (404) 1420 (29.9%)	9860 (2490) 9550 (30.0%)	NC
9 Day 1	500	Mean (SD) Geometric mean (Geo CV%)	2.52 [1.00‐4.00]	1320 (376) 1270 (32.1%)	8340 (2540) 8040 (29.9%)	4.39 (0.38) 4.38 (8.5%)
9 Day 14	500	Mean (SD) Geometric mean (Geo CV%)	1.50 [0.75‐3.00]	1880 (488) 1830 (24.9%)	12,600 (2050) 12,400 (15.8%)	NC

NC, not calculated.

a T_max_ data presented as median [min‐max].

### Pharmacokinetics of RBN‐3143 with Food and Pantoprazole

**Figure 3:**
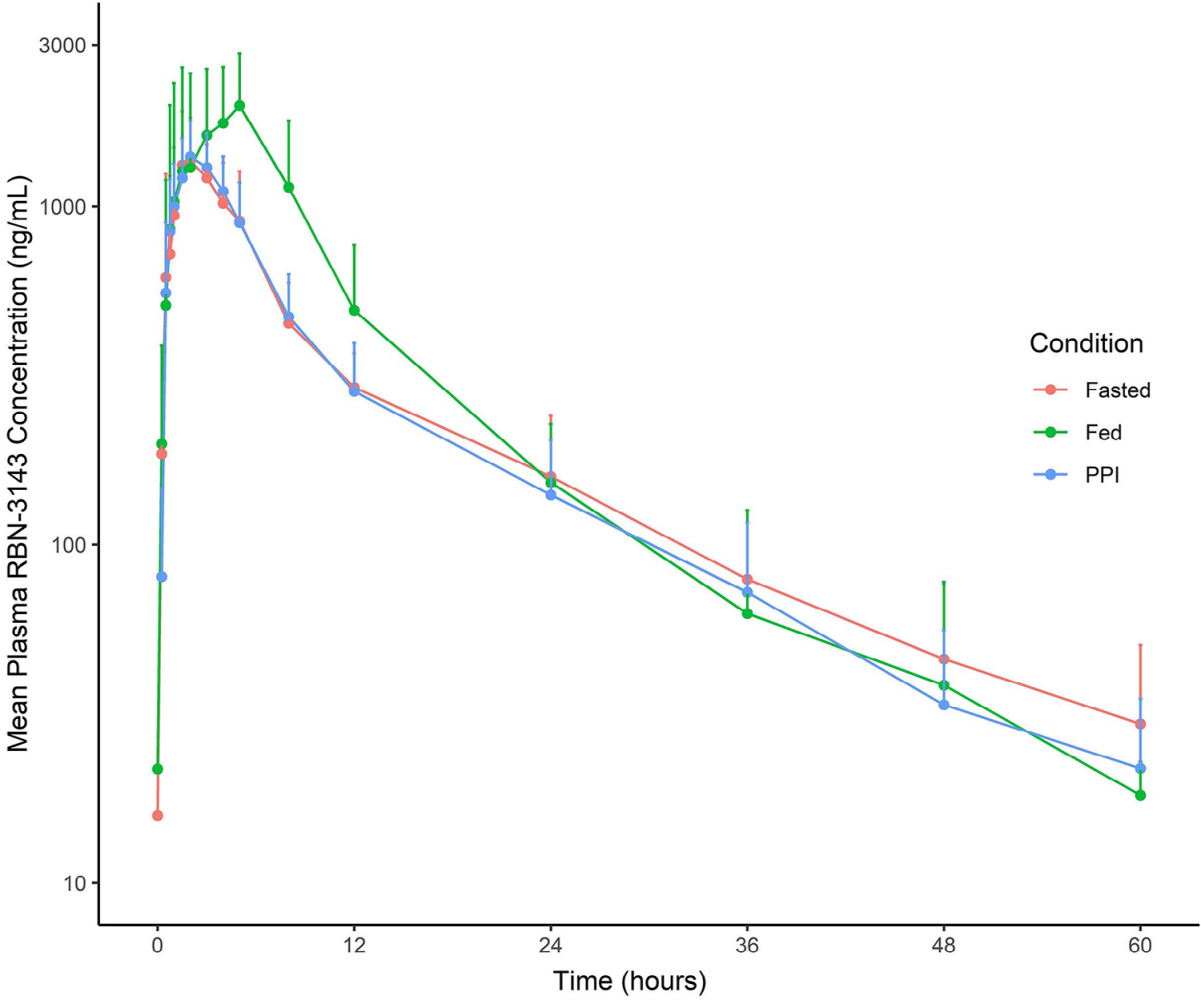
Plasma concentration–time profiles of RBN‐3143 with and without food and following pretreatment with pantoprazole.

As shown in Table 3 and Figure 3, food increased the mean C_max_ and AUC_inf_ of RBN‐3143 by 1.74‐ and 1.42‐fold, respectively, whereas prior treatment with pantoprazole had no effect on RBN‐3143 exposure.

**Table 3:** Statistical Summary and Comparison of RBN‐3143 Pharmacokinetic Parameters Under Fasted and Fed Conditions and After Administration with Pantoprazole

Parameter	Statistics	RBN‐3143 fasted	RBN‐3143 fed	GLSM ratio (90% CI)
C_max_ (ng/mL)	Mean (SD) Geometric mean (Geo CV%) GLSM	1600 (452) 1530 (35.3%) 1548	2780 (802) 2660 (32.1%) 2688	… … 1.74 (1.48, 2.04)
AUC_inf_ (h•ng/mL)	Mean (SD) Geometric mean (Geo CV%) GLSM	13,800 (4000) 13,300 (32.3%) 13,320	19,600 (6060) 18,800 (30.7%) 18,900	… … 1.42 (1.28, 1.58)
AUC_last_ (h•ng/mL)	Mean (SD) Geometric mean (Geo CV%) GLSM	13,300 (3630) 12,800 (31.1%) 12,910	19,400 (6040) 18,600 (30.9%) 18,690	… … 1.45 (1.31, 1.61)

		RBN‐3143 fasted without pantoprazole	RBN‐3143 fasted with pantoprazole	Ratio (90% CI)
C_max_ (ng/mL)	Mean (SD) Geometric mean (Geo CV%) GLSM	1600 (452) 1530 (35.3%) 1529	1580 (360) 1540 (23.9%) 1561	… … 1.02 (0.89, 1.17)
AUC_inf_ (h•ng/mL)	Mean (SD) Geometric mean (Geo CV%) GLSM	13,800 (4000) 13,300 (32.3%) 13,260	13,700 (3150) 13,300 (24.8%) 13,790	… … 1.04 (0.95, 1.14)
AUC_last_ (h•ng/mL)	Mean (SD) Geometric mean (Geo CV%) GLSM	13,300 (3630) 12,800 (31.1%) 12,830	13,400 (3230) 13,000 (26.0%) 13,400	… … 1.04 (0.96, 1.13)

GLSM, geometric least square mean.

Food effect comparison: N = 13 in fasting and fed treatment periods. Median T_max_ after a high‐fat meal was 4 hours compared to 2 hours in the fasted state. Proton pump inhibitor comparison: N = 12 in the RBN‐3143 without pantoprazole and the RBN‐3143 with pantoprazole treatment periods. GLSMs were estimated from a linear mixed‐effects model fitted to the ln‐transformed PK parameters.

### Pharmacokinetic Drug–Drug Interaction of Midazolam with RBN‐3143

**Figure 4:**
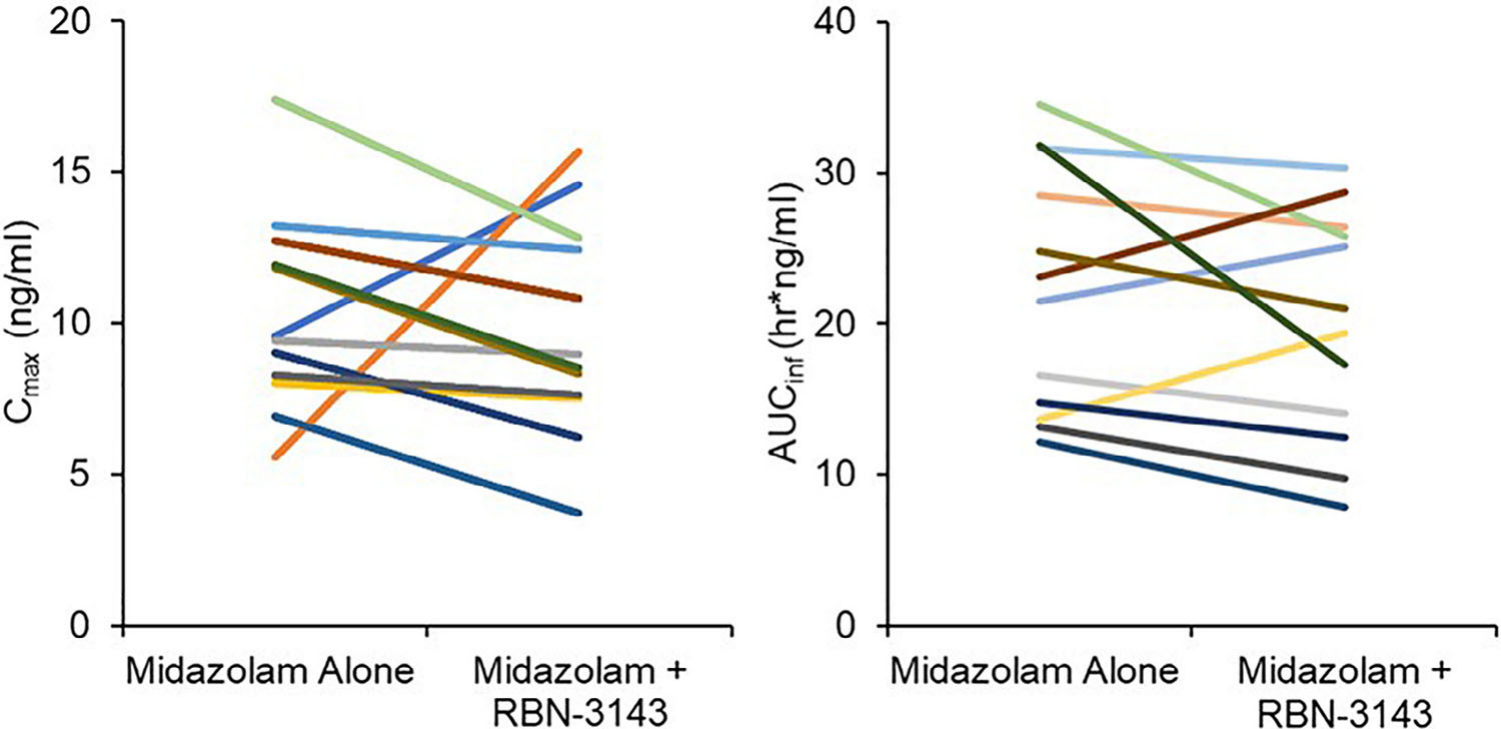
Effects of steady‐state RBN‐3143 dosing on the C_max_ and AUC_inf_ of midazolam in individual subjects.

**Figure 5:**
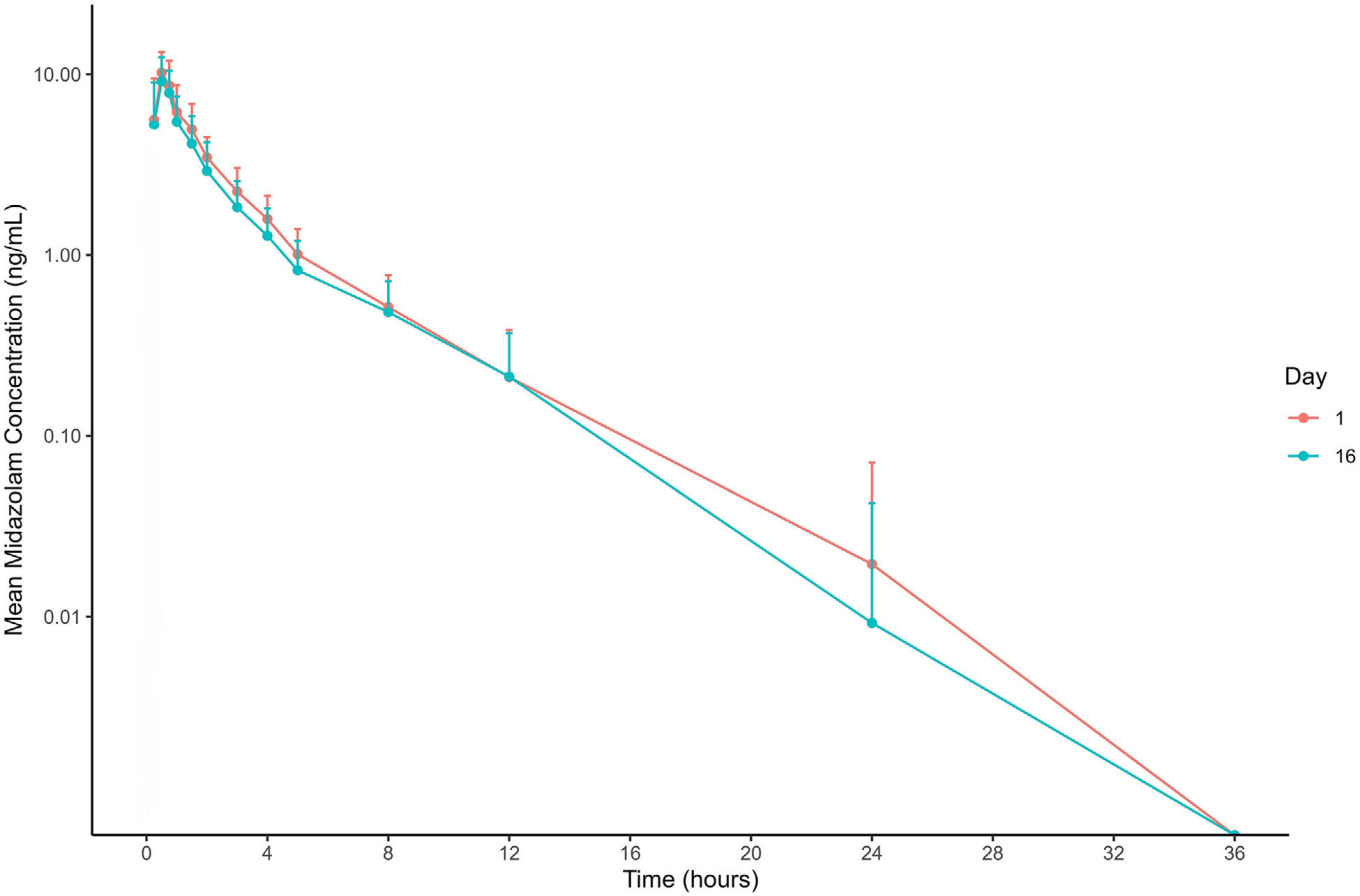
Plasma concentration–time profiles of midazolam with (day 16) and without (day 1) concomitant RBN‐3143 dosed to steady state.

Exposure to oral midazolam was decreased following RNB‐3143 300 mg BID dosing to steady‐state in most subjects (9/12) (Figures 4 and 5), with the mean C_max_ and AUC_inf_ of midazolam decreasing by 0.92‐ and 0.88‐fold, respectively (Table 4).

**Table 4:** Summary of Midazolam Pharmacokinetic Parameters and Statistical Comparison of the Effects of Steady‐State RBN‐3143 Dosing on the Pharmacokinetics of Midazolam

Pharmacokinetic parameters	Statistics	Midazolam 2 mg single dose without RBN‐3143	Midazolam 2 mg single dose with RBN‐3143	GLSM ratio (90% CI)
C_max_ (ng/mL)	Mean (SD) Geometric mean (Geo CV%) GLSM	10.3 (3.24) 9.87 (32.1%) 9.876	9.76 (3.56) 9.11 (42.2%) 9.110	… … 0.92 (0.74, 1.16)
AUC_inf_ (h•ng/mL)	Mean (SD) Geometric mean (Geo CV%) GLSM	22.2 (8.08) 20.8 (39.4%) 20.81	19.9 (7.59) 18.3 (46.5) 18.32	… … 0.88 (0.76, 1.01)
AUC_last_ (h•ng/mL)	Mean (SD) Geometric mean (Geo CV%) GLSM	21.2 (7.87) 19.8 (40.3%) 19.82	18.6 (7.13) 17.2 (46.7%) 17.19	… … 0.87 (0.75, 1.01)
t_1/2_ (hour)	Mean (SD) Geometric mean (Geo CV%)	3.29 (1.41) 3.05 (42%)	3.49 (1.73) 3.09 (56.5%)	

GLSM, geometric least square mean.

RBN‐3143 effect comparison: N = 12 in the midazolam without RBN‐3143 and the midazolam with RBN‐3143 treatment periods. GLSMs were estimated from a linear mixed‐effects model fitted to the ln‐transformed PK parameters.

### Effects of RBN‐3143 on Biomarkers of Renal Function

**Figure 6:**
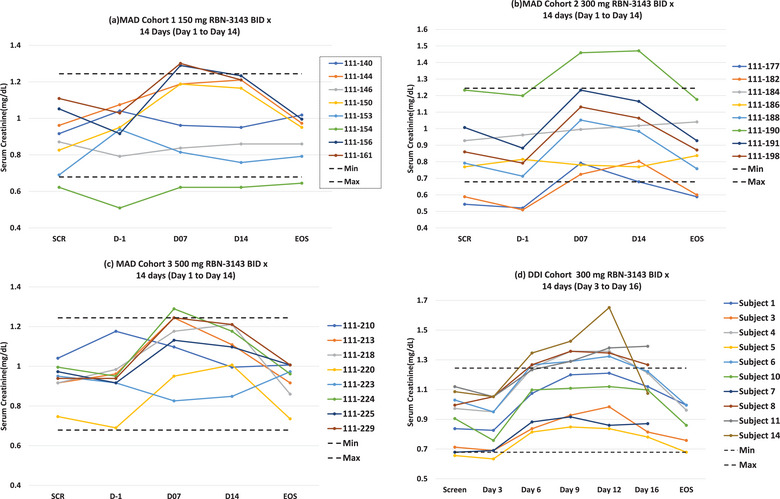
Effects of RBN‐3143 on serum creatinine in individual subjects after twice daily dosing in the MAD cohorts (a, b, and c) and the DDI cohort (d).

When RBN‐3143 was given repeatedly for 14 days in the MAD and DDI cohorts, serum creatinine concentrations consistently increased in most subjects until the end of dosing by 0.1131 to 0.2828 mg/dL from baseline and returned to baseline after dosing ceased (Figure 6). Serum creatinine concentrations stayed below the ULN in 25/34 subjects in total (73.5%) despite elevations from baseline, with a few subjects (9/30, 26.5%) being flagged to the investigators due to elevations above the ULN. There was no evident dose effect, and the placebo‐treated group did not show this trend. Estimated glomerular filtration rates (eGFR) based on serum creatinine consistently fell by 3‐ 9 mL/min/1.83 m^2^ from baseline across all groups taking RBN‐3143 and returned to near baseline on completion. Urea was unchanged, and in the 3‐4 subjects where cystatin C concentrations were obtained, they were stable and renal function using the CKD‐EPI cystatin C equation was unchanged (data not shown).

## Discussion

This is the first human study to evaluate the clinical pharmacology properties of RBN‐3143. Nonclinical work showed that RBN‐3143 is an orally active PARP14 inhibitor. In vitro metabolism data indicated potential for PK‐DDIs as a “victim” via CYP3A4 metabolism and as a “perpetrator” via induction of CYP3A4.

Treatment‐emergent adverse effects occurred with similar frequencies between subjects receiving a single dose of placebo or RBN‐3143 up to 1000 mg and multiple doses of placebo or RBN‐3143 up to 500 mg BID for 14 days. Across all cohorts, the most common treatment‐related TEAEs in subjects taking RBN‐3143 were headache, nausea, vomiting, and increased serum creatinine. Three subjects on RBN‐3143 and 1 subject on placebo had mild increases in thyroid‐stimulating hormone (TSH) without changes in thyroxine (T4) and triiodothyronine (T3) or clinical signs or symptoms of hypothyroidism. The mechanism for these cases of elevated TSH is unknown. Treatment‐related TEAEs were generally mild in severity, indicating that RBN‐3143 was well tolerated. There were no discontinuations in the SAD, MAD, and FE/PPI cohorts for safety reasons. In the DDI cohort, 2 subjects had clinically significant abnormal laboratory findings. One subject had grade 1 elevated serum creatinine, leading to permanent drug discontinuation. Another subject developed grade 2 transaminitis (ALT 3.7 × ULN and AST 1.4 × ULN), the only occurrence of abnormal liver function tests on the study. Both subjects were well. There were no associated AEs, such as changes in blood pressure, urinalyses, or physical examinations, and both AEs resolved within a few days after dosing with RBN‐3143 ceased.

Given the cases of elevated serum creatinine, potential acute kidney injury by RBN‐3143 was investigated by exploring any changes in biomarkers of renal function, including creatinine, urea, and cystatin C (where available).^16^ Serum creatinine increased in all subjects on RBN‐3143 during the first week of dosing without corresponding increases in urea or cystatin C concentrations (where available), with estimates of renal function based on the CKD‐EPI cystatin C equation showing no changes from baseline. Importantly, cystatin C is filtered by the glomeruli, fully reabsorbed, but not actively secreted into renal tubules, giving an estimate of renal function independent of transport processes.^17^ In contrast, creatinine is excreted in urine via glomerular filtration and transporter‐mediated active tubular secretion, the latter contributing between 10% and 40% of total creatinine clearance in healthy adults.^18^, ^19^ The major transporters involved in the active secretion of creatinine are the OAT, OCT, and MATE family members.^20^ A potential mechanism for the elevated serum creatinine is reversible inhibition of efflux transporters by RBN‐3143, but this hypothesis requires testing with in vitro transporter studies and a clinical interaction study using metformin as the MATE probe substrate.^21^ Serum creatinine concentrations returned to baseline 2‐3 days after stopping RBN‐3143, consistent with the elimination of RBN‐3143 from the body, that is, 4‐5 half‐lives is approximately 1‐2 days (Figure 6). Taken together, these data indicate that RBN‐3143 did not cause acute kidney injury. However, clinicians should anticipate mildly elevated serum creatinine concentrations during chronic dosing with RBN‐3143 that are reversible, like the well‐known clinical example of the antibiotic trimethoprim.^22^

The PK of RBN‐3143 was generally dose‐proportional for single doses up to 150 mg and from 300 to 1000 mg. Following BID doses for 14 days, there was a slight accumulation of RBN‐3143 based on C_max_ (1.5‐fold) and AUC (1.6‐fold). Apparent terminal half‐life ranged from about 3 to 11 hours and was generally consistent between doses. Steady‐state was achieved by day 3 of BID dosing (Figure 2B).

When administered with food, the oral absorption of RBN‐3143 was delayed by about 2 hours, and C_max_ and AUC increased by 1.74‐ and 1.42‐fold, respectively (Table 3). Although the exact clinical significance of this data remains unclear until more is learned about the PK/PD relationships of RBN‐3143, it suggests that the food effect should be considered when optimizing the formulation and dosing instructions for RBN‐3143 in further clinical studies. Given that pre‐treatment with pantoprazole did not alter the PK of RBN‐3143, drugs that reduce gastric acid secretion, including H2 blockers and other PPIs that inhibit CYP2C19,^23^ can be taken together with RBN‐3143.

A PK‐DDI cohort with oral midazolam was conducted to determine if RBN‐3143 could cause clinically significant induction of CYP3A4‐mediated drug clearance.^24^, ^25^ In the nonclinical ADME studies, RBN‐3143 induced CYP3A4 mRNA expression in hepatocytes (data not shown). Following 2 weeks of pre‐treatment with RBN‐3143, the mean C_max_ and AUC_inf_ of oral midazolam were decreased by 0.92‐ and 0.88‐fold, respectively (Table 4). However, the induction of CYP3A4 was not universal, with 2 and 3 subjects out of 12 showing increased C_max_ and AUC_inf_, respectively, for midazolam in the presence of RBN‐3143 (Figure 4). Notably, the C_max_ of midazolam in 1 subject increased dramatically against the trend (3‐fold). The exact reason why opposite effects on midazolam C_max_ and AUC_inf_ occurred in some subjects is unknown. Based on the mean data, it would be concluded that clinically significant PK‐DDIs with drugs predominantly cleared by CYP3A4 are unlikely unless the therapeutic index of the “victim” is small and the clinical consequences of slightly altered drug exposure are large, such as with the calcineurin inhibitors (eg, tacrolimus) in transplant medicine^26^ or with estrogen and progestogen containing combined oral contraceptives.^27^

In conclusion, RBN‐3143 was safe with no dose‐limiting toxicities in HVs after single and multiple doses. The PK profile of RBN‐3143 supports BID and possibly QD dosing, depending on PK/PD relationships to be determined in future efficacy studies. Exposure to RBN‐3143 was significantly increased by food but unaffected by pantoprazole, suggesting that RBN‐3143 may be taken with acid‐reducing agents. RBN‐3143 was an inducer of CYP3A4 in most subjects, although this was not universal. Nonetheless, clinically significant PK‐DDIs between RBN‐3143 and drugs predominantly cleared by CYP3A4 are unlikely. Mild reversible increases in serum creatinine occur with RBN‐3143, possibly due to inhibition of active tubular creatinine secretion. RBN‐3143 did not cause acute kidney injury. Future clinical studies are warranted to evaluate RBN‐3143 in the treatment of inflammatory diseases with pathological TH2/TH17‐cytokine signaling.

## Conflicts of Interest

T.M.P., B.M.J., and K.J.P. are employees of CMAX Clinical Research Pty Ltd, which was contracted by Ribon Therapeutics Inc. to conduct this study. V.B., E.M., J.N., B.Y., S.P., and V.S. were employees of Ribon Therapeutics Inc. during the conduct of this study.

## Funding

This study was sponsored by Ribon Therapeutics Inc.

## Supporting information



Figure S1

Supporting Tables

## Data Availability

Original data files may be made available upon request.

## References

[1] Cohen MS , Chang P . Insights into the biogenesis, function, and regulation of ADP‐ribosylation. Nat Chem Biol. 2018;14(3):236‐243. doi:10.1038/nchembio.2568 29443986 PMC5922452

[2] Lüscher B , Ahel I , Altmeyer M , et al. ADP‐ribosyltransferases, an update on function and nomenclature. FEBS J. 2022;289(23):7399‐7410. doi:10.1111/febs.16142 34323016 PMC9027952

[3] Goenka S , Boothby M . Selective potentiation of Stat dependent gene expression by collaborator of Stat6 (CoaSt6), a transcriptional factor. Proc Natl Accad Sci USA. 2006;102(11):4210‐4215. 10.1073/pnas.0506981103 PMC1449672 16537510

[4] Mehrotra P , Krishnamurthy P , Sun J , Goenka S , Kaplan MH . Poly‐ADP‐ribosyl polymerase‐14 promotes T helper 17 and follicular T helper development. Immunology. 2015;146(4):537‐546. doi:10.1111/imm.12515 26222149 PMC4693893

[5] He H , Bissonnette R , Wu J , et al. Tape strips detect distinct immune and barrier profiles in atopic dermatitis and psoriasis. J Allergy Clin Immunol. 2021;147(1):199‐212. doi:10.1016/j.jaci.2020.05.048 32709423

[6] Yick CY , Zwinderman AH , Kunst PW , et al. Transcriptome sequencing (RNA‐Seq) of human endobronchial biopsies: asthma versus controls. Eur Respir J. 2013;42(3):662‐670. doi:10.1183/09031936.00115412 23314903

[7] Iwata H , Goettsch C , Sharma A , et al. PARP9 and PARP14 cross‐regulate macrophage activation via STAT1 ADP‐ribosylation. Nat Comfmun. 2016;7:12849. doi:10.1038/ncomms12849 PMC5095532 27796300

[8] Schenkel LB , Molina JR , Swinger KK , et al. A potent and selective PARP14 inhibitor decreases protumor macrophage gene expression and elicits inflammatory responses in tumor explants. Cell Chem Biol. 2021;28(8):1158‐1168.e13. doi:10.1016/j.chembiol.2021.02.010 33705687

[9] Cho SH , Raybuck A , Wei M , et al. B cell‐intrinsic and ‐extrinsic regulation of antibody responses by PARP14, an intracellular (ADP‐ribosyl)transferase. J Immunol. 2013;191(6):3169‐3178. doi:10.4049/jimmunol.1301106 23956424 PMC3770464

[10] Mehrotra P , Hollenbeck A , Riley JP , et al. Poly (ADP‐ribose) polymerase 14 and its enzyme activity regulates T(H)2 differentiation and allergic airway disease. J Allergy Clin Immunol. 2013;131(2):521‐531.e1‐12. doi:10.1016/j.jaci.2012.06.015 22841009 PMC3502685

[11] Lyly A , Laulajainen‐Hongisto A , Gevaert P , Kauppi P , Toppila‐Salmi S . Monoclonal antibodies and airway diseases. Int J Mol Sci. 2020;21(24):9477. doi:10.3390/ijms21249477 33322143 PMC7763928

[12] Ahn J , Choi Y , Simpson EL . Therapeutic new era for atopic dermatitis: part 1. Biologics. Ann Dermatol. 2021;33(1):1‐10. doi:10.5021/ad.2021.33.1.1 33911806 PMC7875213

[13] Ahn J , Choi Y , Simpson EL . Therapeutic new era for atopic dermatitis: part 2. Small molecules. Ann Dermatol. 2021;33(2):101‐107. doi:10.5021/ad.2021.33.2.101 33935450 PMC8082001

[14] Kurokawa N , Robinson MK , Bernard C , et al. Safety and immunogenicity of a plant‐derived rotavirus‐like particle vaccine in adults, toddlers and infants. Vaccine. 2021;39(39):5513‐5523. doi:10.1016/j.vaccine.2021.08.052 34454786

[15] Polasek TM , Leelasena I , Betscheider I , et al. Safety, tolerability, and pharmacokinetics of IMU‐935, a novel inverse agonist of retinoic acid receptor‐related orphan nuclear receptor gammat: results from a double‐blind, placebo‐controlled, first‐in‐human phase 1 study. Clin Pharmacol Drug Dev. 2023;12(5):525‐534. doi:10.1002/cpdd.1243 36938862

[16] Binnenmars SH , Hijmans RS , Navis G , de Borst MH . Biomarkers of renal function: towards clinical actionability. Clin Pharmacol Ther. 2017;102(3):481‐492. doi:10.1002/cpt.765 28608458

[17] Nyman U , Bjork J , Back SE , Sterner G , Grubb A . Estimating GFR prior to contrast medium examinations—what the radiologist needs to know! Eur Radiol. 2016;26(2):425‐435. doi:10.1007/s00330-015-3842-9 26017739

[18] Breyer MD , Qi Z . Better nephrology for mice–and man. Kidney Int. 2010;77(6):487‐489. doi:10.1038/ki.2009.544 20186162

[19] Levey AS , Perrone RD , Madias NE . Serum creatinine and renal function. Ann Rev Med. 1988;39:465‐490. 3285786 10.1146/annurev.me.39.020188.002341

[20] Chu X , Bleasby K , Chan GH , Nunes I , Evers R . The complexities of interpreting reversible elevated serum creatinine levels in drug development: does a correlation with inhibition of renal transporters exist? Drug Metab Dispos. 2016;44(9):1498‐1509. doi:10.1124/dmd.115.067694 26825641

[21] Trueck C , Hsin CH , Scherf‐Clavel O , et al. A clinical drug‐drug interaction study assessing a novel drug transporter phenotyping cocktail with adefovir, sitagliptin, metformin, pitavastatin, and digoxin. Clin Pharmacol Ther. 2019;106(6):1398‐1407. doi:10.1002/cpt.1564 31247117

[22] Delanaye P , Mariat C , Cavalier E , Maillard N , Krzesinski JM , White CA . Trimethoprim, creatinine and creatinine‐based equations. Nephron Clin Pract. 2011;119(3):c187‐c194. doi:10.1159/000328911 21832843

[23] Ogilvie BW , Yerino P , Kazmi F , et al. The proton pump inhibitor, omeprazole, but not lansoprazole or pantoprazole, is a metabolism‐dependent inhibitor of CYP2C19: implications for coadministration with clopidogrel. Drug Metab Dispos. 2011;39(11):2020‐2033. doi:10.1124/dmd.111.041293 21795468

[24] Polasek TM , Lin FKL , Miners JO , Doogue MP . Perpetrators of pharmacokinetic drug–drug interactions from altered cytochrome P450 activity: a criteria‐based assessment. Br J Clin Pharmacol. 2011;71(5):727‐736. doi:10.1111/j.1365-2125.2011.03903.x. 21223357 PMC3093078

[25] Snyder BD , Rowland A , Polasek TM , Miners JO , Doogue MP . Evaluation of felodipine as a potential perpetrator of pharmacokinetic drug–drug interactions. Eur J Clin Pharmacol. 2014;70(9):1115‐1122. doi:10.1007/s00228-014-1716-8 25028073

[26] Størset E , Asberg A , Skauby M , et al. Improved tacrolimus target concentration achievement using computerized dosing in renal transplant recipients – a prospective, randomized study. Transplantation. 2015;99(10):2158‐2166. doi:10.1097/TP.0000000000000708 25886918 PMC4591080

[27] Zhang N , Shon J , Kim MJ , et al. Role of CYP3A in oral contraceptives clearance. Clin Transl Sci. 2018;11(3):251‐260. doi:10.1111/cts.12499 28986954 PMC5944580

