# Supplementary figures and images for: First‐in‐Human Phase 1 Study to Evaluate the Clinical Pharmacology Properties of RBN‐3143, a Novel Inhibitor of Mono‐Adenosine Diphosphate Ribosyltransferase‐PARP14
Source: Clin Pharmacol Drug Dev. 2025 Apr 30;14(7):493–504. doi: 10.1002/cpdd.1539 (PMC12209991; doi:10.1002/cpdd.1539)

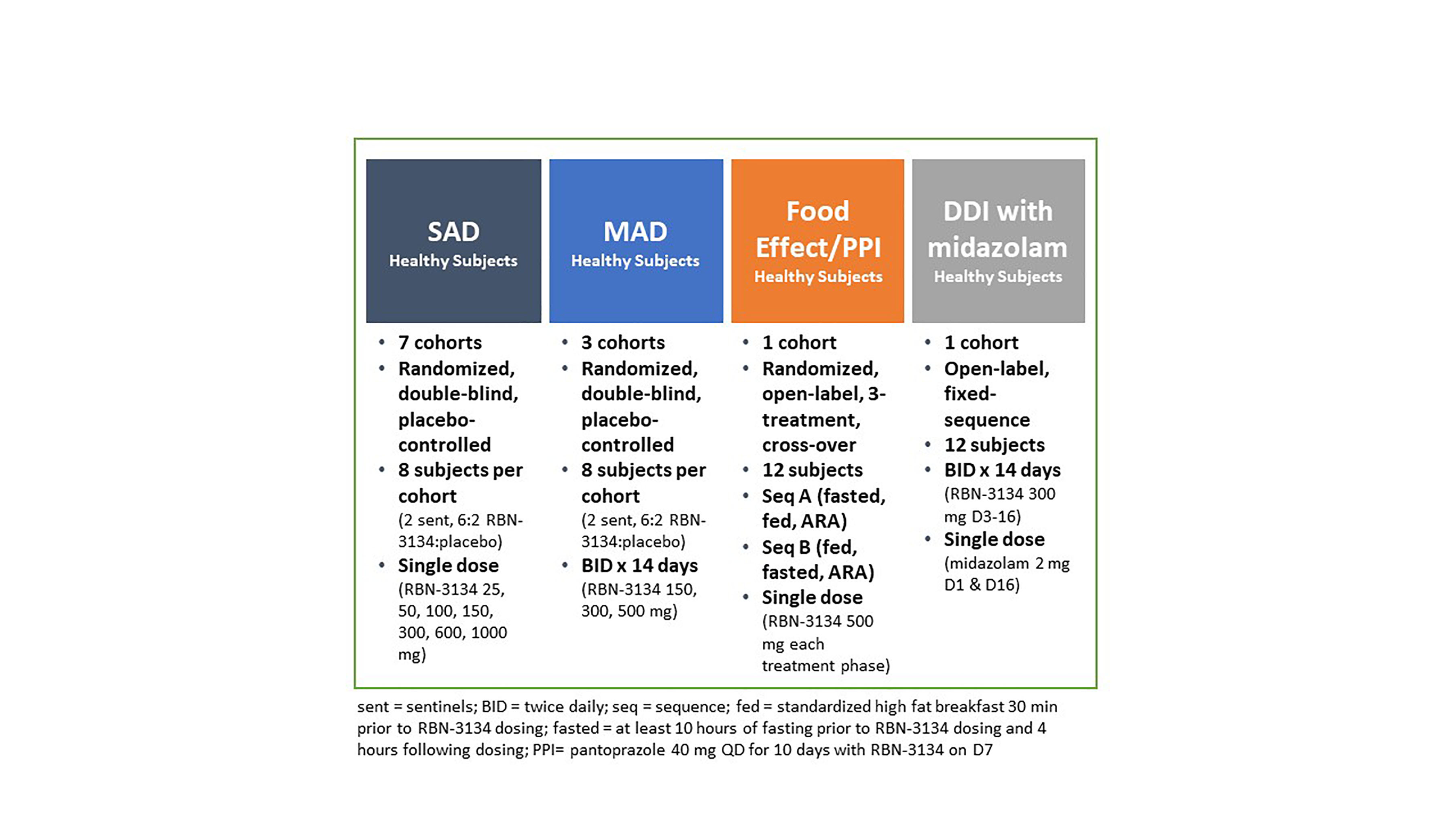

Supplement: Supplementary file 1 — Figure S1 [file CPDD-14-493-s002.jpeg]
