# Supplementary material for: First‐in‐Human Phase 1 Study to Evaluate the Clinical Pharmacology Properties of RBN‐3143, a Novel Inhibitor of Mono‐Adenosine Diphosphate Ribosyltransferase‐PARP14
Source: Clin Pharmacol Drug Dev. 2025 Apr 30;14(7):493–504. doi: 10.1002/cpdd.1539 (PMC12209991; doi:10.1002/cpdd.1539)
Supplement: Supplementary file 2 — Supporting Tables [file CPDD-14-493-s001.docx]

Table S1. Inclusion and exclusion criteria.

| **Inclusion criteria** |
| --- |
| 1. Healthy male or female subjects between 18 and 65 years of age (inclusive at the time of informed consent) |
| 2. The subject must have a body mass index (BMI) between ≥ 18 and ≤ 35 kg/m2 (inclusive) at Screening |
| 3. The subject must be in good general health, with no significant medical history, have no clinically significant abnormalities on physical examination (in the opinion of the Investigator) at Screening and/or before administration of the initial dose of study drug |
| 4. The subject must have clinical laboratory values within normal range as specified by the testing laboratory, unless deemed not clinically significant by the Investigator or delegate |
| 5. The subject is willing and able to speak, read, and understand English, and provide written informed consent after the nature of the study has been explained and prior to the commencement of any study procedures |
| 6. The subject must be a non-smoker or former smoker. If a former smoker, the subject must not have used in excess of 5 tobacco/nicotine containing products per month within the 3 months prior to Screening. Subjects must have negative cotinine results in drug tests at Screening and Baseline |
| 7. Females must be:  • Non-pregnant  • Non-lactating  • Must use a non-hormonal, acceptable, highly effective double contraception from Screening until study completion through EOS.  Acceptable non-hormonal double contraception is defined as a condom AND one other form of the following:  − An IUD (non-hormonal)  − Documented evidence of surgical sterilization at least 6 months prior to Screening (eg, tubal occlusion, hysterectomy, bilateral salpingectomy, or bilateral oophorectomy for women or vasectomy for men [with appropriate post-vasectomy documentation of the absence of sperm in semen] provided the male partner is a sole partner)  WOCBP must have:  • A negative pregnancy test at Screening and Day -1 and be willing to have additional pregnancy tests as required throughout the study  Postmenopausal Women:  Women not of childbearing potential must be:  • Post-menopausal for ≥ 12 months  • Postmenopausal status will be confirmed through testing of FSH levels ≥ 40 IU/L at Screening for amenorrhoeic female subjects  • Postmenopausal women who are currently taking and are compliant with hormone replacement therapy ≥ 6 months are eligible regardless of FSH level  Abstinence:  • Females who are abstinent from heterosexual intercourse will also be eligible. Complete abstinence by the subject for the duration of the study through EOS. Note: Periodic abstinence (eg, calendar, ovulation, symptothermal, post-ovulation methods) and withdrawal are not considered highly effective methods of birth control.  • Female subjects who are in same-sex relationships are not required to use contraception.  Males:  If engaged in sexual relations with a WOCBP:  • The subject or his partner must be surgically sterile (eg, > 30 days since vasectomy with no viable sperm tubal occlusion, hysterectomy, bilateral salpingectomy, bilateral oophorectomy)  • Must use an acceptable, highly effective contraceptive method from Screening until study completion through EOS. Acceptable methods of contraception include the use of condoms and the use of an effective contraceptive for the female partner that includes:  − OCPs  − Long-acting implantable hormones  − Injectable hormones  − Vaginal ring  − Use of an IUD  • Subjects with same-sex partners (abstinence from penile-vaginal intercourse) are eligible |
| 8. For at least 90 days after the last dose of study drug, male subjects must not donate sperm and female subjects must not donate ova |
| 9. The subject must agree to comply with the drawing of blood samples for the PK assessments |
| 10. The subject must be willing and able to attend the necessary visits to the CRU, and comply with all testing, fasting and requirements defined in the protocol |
| 11. The subject must be willing and able to remain at the study site unit for the duration ofthe confinement period and return for the outpatient visit/s defined in the protocol |
| **Exclusion criteria** |
| 1. Any medical condition that is considered by the Investigator or delegate that may interfere with study assessments, may adversely affect the patient’s participation in the study, may make the patient’s participation in the study unreliable, or be of such severity as to present an increased risk to the patient because of participation in the study. Justification for exclusion under this criterion will be documented in study documents (CRF) |
| 2. Prior or ongoing medical conditions, physical findings, laboratory abnormality or a history of neurological, hepatic, renal, endocrine, including thyroid, cardiovascular, gastrointestinal, pulmonary, or metabolic disease that is considered as significant by the Investigator or delegate, and in the Investigator’s (or delegate’s) opinion, could adversely affect the safety of the subject |
| 3. Abnormal ECG findings at Screening that are considered by the Investigator to be clinically significant:  a. Significant history of cardiovascular disease, as judged by the Investigator.  b. ECG results showing QTcF > 450 msec or the presence of clinically significant abnormalities as determined by the Investigator (Screening or Day -1).  c. Elevation of blood pressure (BP), ie. semi-recumbent or supine systolic BP > 150 mmHg and/or diastolic BP > 90 mmHg, or heart rate > 100 beats per minute at rest (Screening or Day -1) |
| 4. Use of any prescription medication within 14 days of dosing or over-the-counter (OTC) medication (including vitamins) within 48 hours of dosing or intends to use any prescription medication or OTC medication during the study that may interfere with the evaluation of study medication. The co-administration of medications known to have high risk of prolonging the QT interval are also prohibited. The use of other concomitant medications that present a low risk of QT prolongation may be considered, with the approval of the Medical Monitor. Simple analgesia (paracetamol, nonsteroidal anti-inflammatory drug [NSAID]) may be permitted at the discretion of the Investigator or delegate. Use of a low dose inhaled corticosteroid and β2 agonist inhalers to treat concomitant Asthma are allowed |
| 5. Ingestion of herbal medicines within 3 weeks before Screening, and grapefruit, grapefruit juice, star fruit or orange marmalade (made with Seville oranges) within 2 weeks prior to dosing, or intends to use any of these products during the study |
| 6. Subjects must have no relevant dietary restrictions, and be willing to consume standard meals provided |
| 7. Subjects to abstain from ingesting caffeine- or xanthine-containing products (eg. coffee, tea, cola drinks, and chocolate) on days scheduled for full PK sample collection from 10 hours prior to the start of dosing through 12 hours postdose; at other times, caffeine- and other (methyl)xanthine-containing foods and beverages may be served (or withheld, as applicable) in accordance with normal study center policy. Subjects will be required to refrain from consumption of alcohol from the day prior to the days of PK sample collection through to 12 hours postdose; at other times light alcohol consumption is permitted. Light alcohol consumption is defined as not more than 1 unit of alcohol per day, equivalent to 1 bottle of beer (350 mL), or 1 glass of wine (150 mL), or 1 liquor drink (40 mL) per day |
| 8. A history of substance abuse or dependency or history of recreational intravenous (IV) drug use over the last 1 year (by self-declaration); or a positive ethanol breath test, urine cotinine, or urine drug screen at Screening and at Day -1 |
| 9. A positive test for hepatitis C antibody (HCV), hepatitis B surface antigen (HBsAg) and/or human immunodeficiency virus (HIV) antibody. A positive tuberculosis (TB) test (if performed as per local institutional guidelines and at the Investigator’s discretion) and a positive COVID-19 (if conducted, at the Investigator’s discretion) at Screening |
| 10. Pregnant or lactating at Screening or planning to become pregnant (self or partner) at any time during the study, including the Follow-up Period |
| 11. Use of any investigational product (IP) or investigational medical device within 30 days prior to Screening, or 5 half-lives of the product (whichever is the longest) or participation in more than 4 investigational drug studies within 1 year prior to Screening |
| 12. Donated or lost a significant volume of blood (> 450 mL) within 4 weeks prior to the first study drug administration |
| 13. Unwilling to reside in the study unit for the duration of the study or to cooperate fully with the Investigator, delegate or site personnel |
| 14. Alkaline phosphatase (ALP), aspartate aminotransferase (AST), alanine aminotransferase (ALT) and/or total bilirubin >1.5 × upper limit of normal at Screening. Repeat testing at Screening is acceptable for out-of-range values following approval by the Investigator or delegate |
| 15. Estimated Glomerular Filtration Rate (eGFR) < 90 mL/min/1.73 m2 at Screening |
| 16. Presence of any underlying physical or psychological medical condition that, in the opinion of the Investigator, would make it unlikely that the subject will comply with the protocol or complete the study per protocol |
| 17. Plasma donation within 7 days prior to the first study drug administration |
| 18. Fever (oral temperature >38°C) or symptomatic viral or bacterial infection within 2 weeks prior to Day -1 |
| 19. History of severe allergic or anaphylactic reactions to medicines or vaccines |
| 20. History of malignancy, except for non-melanoma skin cancer, treated or excised more than 2 years ago and cervical intraepithelial neoplasia that has been successfully cured more than 5 years prior to Screening |
| 21. History or presence of a condition associated with significant immunosuppression |
| 22. History of life-threatening infection (eg, meningitis) |
| 23. Infections requiring parenteral antibiotics within the 6 months prior to Screening |
| 24. For the Food effect cohort only: a diet that in the opinion of the Investigator is incompatible with the on-study diet (eg, lactose intolerance diet) |
| 25. Unwilling to refrain from strenuous exercise within 48 hours prior to visits and during confinement at the CRU |

Table S2. Schedules of assessments.

Schedule of Assessments – Single Ascending Dose (SAD)

|  | Screening | Treatment Perioda | | | | | EOS/Follow-up Visitb | ET Visitc |
| --- | --- | --- | --- | --- | --- | --- | --- | --- |
| Study Day | -28 to -2 | -1 | 1 | 2 | 3 | 4 | 7 ± 1 | - |
| Study visit (CRU) | X | X | | | | | X | X |
| Informed consent | X |  |  |  |  |  |  |  |
| Demographics | X |  |  |  |  |  |  |  |
| Medical history/ concurrent conditions | X |  |  |  |  |  |  |  |
| Smoking history | X | X |  |  |  |  |  |  |
| Inclusion/exclusion criteriad | X | X | X |  |  |  |  |  |
| Randomization |  |  | X |  |  |  |  |  |
| 12-lead ECGe | X | X | X | X |  |  | X | X |
| Physical examinationf | X | X |  |  |  |  | X | X |
| Height/weight/BMIg | X | X |  |  |  |  | X | X |
| Holter monitoringh |  |  | X | X |  |  |  |  |
| Vital signsi | X | X | X | X | X | X | X | X |
| Drugs of abuse/alcoholj | X | X |  |  |  |  |  |  |
| Virologyk | X |  |  |  |  |  |  |  |
| Pregnancy test (WOCBP only)l | X | X |  |  |  |  | X | X |
| FSH test (postmenopausal women only) | X |  |  |  |  |  |  |  |

Schedule of Assessments – Single Ascending Dose (SAD) (Continued)

|  | Screening | Treatment Perioda | | | | | EOS/Follow-up Visitb | ET Visitc |
| --- | --- | --- | --- | --- | --- | --- | --- | --- |
| Study Day | -28 to -2 | -1 | 1 | 2 | 3 | 4 | 7 ± 1 | - |
| Clinical laboratory tests (blood and urine)m | X | X |  |  |  |  | X | X |
| Thyroid function testing^n^ |  | X |  | X |  |  | X | X |
| Study drug administration^o^ |  |  | X |  |  |  |  |  |
| Blood sample for PK^p^ |  |  | X | X | X | X | X^q^ | X^q^ |
| Urine sample for PK^r^ |  |  | X | X |  |  |  |  |
| Blood sample for PD^s^ |  |  | X | X |  |  |  |  |
| Prior and concomitant medications | X | X | X | X | X | X | X | X |
| Adverse events | X | X | X | X | X | X | X | X |

Abbreviations: anti-TG = anti-thyroglobulin antibody; anti-TPO = anti-thyroid peroxidase antibody; BMI = body mass index; CRU = clinical research unit; ECG = electrocardiogram; ET = early termination; EOS = end of study; FSH = follicle stimulating hormone; fT3 = free triiodothyronine; fT4 = free thyroxine; PD = pharmacodynamics; PK = pharmacokinetics; TSH = thyroid-stimulating hormone; WOCBP = women of childbearing potential.

a Subjects will be required to remain in the CRU from Day -1 until completion of scheduled assessments on Day 4.

b Subjects will be required to return to the CRU for an EOS/Follow-up visit at 5 to 7 days following study drug administration (ie, Day 7 ± 1).

c Subjects who withdraw early or are discontinued from the study will be encouraged to return to the CRU for an Early Termination visit (EOS assessment).

d Eligibility will be confirmed at Screening, Day -1/prior to dosing on Day 1.

e Triplicate ECGs (each reading taken at least 1 minute apart) will be taken at Screening and prior to dosing on Day 1, to establish baseline. All other ECGs (Day -1 and postdose) will be single readings unless repeat ECG is indicated. ECGs will be taken at Screening, Day -1 and within 1 hour predose, 1, 2, 4 hours (Day 1), 24 hours postdose (Day 2), and at the EOS/Follow-up or ET visit. ECGs will be taken after the subject has rested for approximately 10 minutes in a semi-recumbent or supine position. ECGs should be taken within ±15 minutes of the nominal timepoint. Where assessments coincide, vital signs should be conducted first, followed by ECGs, then blood draws for PK/safety labs.

f Complete physical examination required at Screening, Day -1 and the EOS/Follow-up or ET visit in all cohorts. A symptom-directed physical examination will otherwise be performed as clinically indicated on all other days.

g At the Screening visit, height, weight and BMI measurements are required. On Day -1, weight and BMI measurements are required. At the EOS/Follow-up or ET Visit, only weight is required to be measured.

h Continuous Holter monitoring will be conducted in each of the SAD cohorts, starting from at least 1 hour predose and until at least 24 hours postdose. 12-lead ECGs will be extracted from the continuous recording by the central ECG laboratory at appropriate time points matching the PK collection.

i Vital signs (pulse rate, systolic and diastolic blood pressure, respiratory rate and aural [tympanic] temperature) will be measured at Screening, Day -1, within 1 hour predose on Day 1, and at the following postdose timepoints: 1, 2, 3, 4, 6, 8, 10, 12 hours (Day 1), 24 hours postdose (Day 2), 48 hours postdose (Day 3), 72 hours postdose (Day 4), and at the EOS/Follow-up visit or ET visit. Vital signs will be taken after approximately 5 minutes rest in a semi-recumbent or supine position. Where assessments coincide, vital signs should be conducted first, followed by ECGs, then blood draws for PK/safety labs.

j Urine drug screen (including cotinine) and ethanol breath test to be performed.

k Includes testing for hepatitis C antibody (HCV), hepatitis B surface antigen (HbsAg), human immunodeficiency virus (HIV) antibody. May also include testing for COVID-19 (using a locally approved test) at the Investigator’s discretion.

l For WOCBP, a serum pregnancy test is to be performed at the Screening visit, and a urine pregnancy test will be performed at all other timepoints as indicated.

^m^ Subjects are required to fast for at least 4 hours prior to collection of blood samples for biochemistry, hematology and coagulation analysis, and a urine sample for urinalysis. On Day 1, samples are to be collected prior to dosing.

^n^ Thyroid function testing, TSH, fT3, fT4 anti-TG and anti-TPO taken at Day -1, at 24 hours postdose (Day 2) and at the EOS or ET.

^o^ For Part A SAD, the criteria for the fasted state will be at least 10 hours overnight prior to dosing, which is to be continued for 4 hours following dose administration.

^p^ Blood samples for PK assessments will be taken at the timepoints described in Table S3.

^q^ An additional blood sample for PK analysis may be collected at EOS/Follow-up or ET visit, if appropriate.

^r^ A urine sample for the measurement of RBN-3143 in urine will be collected predose on Day 1. Pooled urine collections will be taken at the timepoints described in Table S3.

^s^ Blood samples for PD assessments will be taken predose on Day 1, and at 2, 8, and 24 hours postdose.

Schedule of Assessments – Multiple Ascending Dose (MAD)

|  | Screening | Treatment Perioda | | | | | | | | | | | | | | | | EOS/ Follow-upb | ET Visitc |
| --- | --- | --- | --- | --- | --- | --- | --- | --- | --- | --- | --- | --- | --- | --- | --- | --- | --- | --- | --- |
| Study Days | -28 to -2 | -1 | 1 | 2 | 3 | 4 | 5 | 6 | 7 | 8 | 9 | 10 | 11 | 12 | 13 | 14 | 15 | 20 ± 1 | - |
| Study visit (CRU) | X | X | | | | | | | | | | | | | | | | X | X |
| Informed consent | X |  |  |  |  |  |  |  |  |  |  |  |  |  |  |  |  |  |  |
| Demographics | X |  |  |  |  |  |  |  |  |  |  |  |  |  |  |  |  |  |  |
| Medical history/concurrent conditions | X |  |  |  |  |  |  |  |  |  |  |  |  |  |  |  |  |  |  |
| Smoking history | X | X |  |  |  |  |  |  |  |  |  |  |  |  |  |  |  |  |  |
| Inclusion/exclusion criteriad | X | X | X |  |  |  |  |  |  |  |  |  |  |  |  |  |  |  |  |
| Randomization |  |  | X |  |  |  |  |  |  |  |  |  |  |  |  |  |  |  |  |
| 12-lead ECGe | X | X | X |  |  |  |  |  | X |  |  |  |  |  |  | X |  | X | X |
| Physical examinationf | X | X |  |  |  |  |  |  |  |  |  |  |  |  |  |  |  | X | X |
| Height/weight/BMIg | X | X |  |  |  |  |  |  |  |  |  |  |  |  |  |  |  | X | X |
| Holter monitoringh |  |  | X |  |  |  |  |  |  |  |  |  |  |  |  | X |  |  |  |
| Vital signsi | X | X | X | X | X | X | X | X | X | X | X | X | X | X | X | X | X | X | X |
| Drugs of abuse/alcoholj | X | X |  |  |  |  |  |  |  |  |  |  |  |  |  |  |  |  |  |
| Virologyk | X |  |  |  |  |  |  |  |  |  |  |  |  |  |  |  |  |  |  |
| Pregnancy test (WOCBP only)l | X | X |  |  |  |  |  |  |  |  |  |  |  |  |  |  |  | X | X |
| FSH test (postmenopausal women only) | X |  |  |  |  |  |  |  |  |  |  |  |  |  |  |  |  |  |  |

Schedule of Assessments – Multiple Ascending Dose (MAD) (Continued)

|  | Screening | Treatment Perioda | | | | | | | | | | | | | | | | EOS/  Follow-upb | ET Visitc |
| --- | --- | --- | --- | --- | --- | --- | --- | --- | --- | --- | --- | --- | --- | --- | --- | --- | --- | --- | --- |
| Study Days | -28 to -2 | -1 | 1 | 2 | 3 | 4 | 5 | 6 | 7 | 8 | 9 | 10 | 11 | 12 | 13 | 14 | 15 | 20 ± 1 | - |
| Clinical laboratory tests (blood and urine)m | X | X |  |  |  |  |  |  | X |  |  |  |  |  |  | X |  | X | X |
| Thyroid function testing^n^ |  | X |  | X |  |  |  |  | X |  |  |  |  |  |  | X |  | X | X |
| Study drug administrationo |  |  | X^p^ | X^p^ | X^p^ | X^p^ | X^p^ | X^p^ | X^p^ | X^p^ | X^p^ | X^p^ | X^p^ | X^p^ | X^p^ | X^p^ |  |  |  |
| Blood sample for PK^q^ |  |  | X | X | X | X | X | X | X | X | X | X | X | X | X | X |  | X | X |
| Urine sample for PK_r_ |  |  | X |  |  |  |  |  |  |  |  |  |  |  |  | X |  |  |  |
| Blood sample for PD^s^ |  |  | X |  |  |  |  |  | X |  |  |  |  |  |  | X |  |  |  |
| Prior and concomitant medications | X | X | X | X | X | X | X | X | X | X | X | X | X | X | X | X | X | X | X |
| Adverse events | X | X | X | X | X | X | X | X | X | X | X | X | X | X | X | X | X | X | X |
| Abbreviations: anti-TG = anti-thyroglobulin antibody; anti-TPO = anti-thyroid peroxidase antibody; BMI = body mass index; CRU = clinical research unit; ECG = electrocardiogram; ET = early termination; EOS = end of study; FSH = follicle-stimulating hormone; fT3 = free triiodothyronine; fT4 = free thyroxine; PD = pharmacodynamics; PK = pharmacokinetics; TSH = thyroid-stimulating hormone; WOCBP = women of childbearing potential. | | | | | | | | | | | | | | | | | | | |

a Subjects will be required to remain in the CRU from Day -1 until completion of scheduled assessments on Day 15.

b Subjects will be required to return to the CRU for an EOS/Follow-up visit at 5 to 7 days after the final dose of study drug (ie, Day 20 ±1 day).

c Subjects who withdraw early or are discontinued from the study will be encouraged to return to the CRU for an Early Termination visit (EOS assessment).

d Eligibility will be confirmed at Screening, Day -1/prior to dosing on Day 1.

e Triplicate ECGs (each reading taken at least 1 minute apart) will be taken at Screening and prior to dosing on Day 1, to establish baseline. All other ECGs (Day -1 and postdose) will be single readings unless repeat ECG is indicated. ECGs will be taken at Screening, Day -1, within 1 hour predose and at 1, 2, 4 hours postdose (Day 1 and Day 14). 12-lead ECGs will also be taken within 1 hour predose and 2 hours postdose on Day 7, and at the EOS/Follow-up or ET visit. ECGs will be taken after the subject has rested for approximately 10 minutes in a semi-recumbent or supine position. Where assessments coincide, vital signs should be conducted first, followed by ECGs, then blood draws for PK/safety labs.

f Complete physical examination required at Screening, Day -1 and the EOS/Follow-up or ET visit in all cohorts. A symptom-directed physical examination will otherwise be performed as clinically indicated on all other days.

g At the Screening visit, height, weight and BMI measurements are required. On Day -1, weight and BMI measurements are required. At the EOS/Follow-up or ET Visit, only weight is required to be measured.

h Continuous Holter monitoring will be conducted from at least 1 hour predose until at least 12 hours postdose on Day 1 and Day 14. 12-lead ECGs will be extracted from the continuous recording by the central ECG laboratory at appropriate time points matching the PK collection.

i Vital signs (pulse rate, systolic and diastolic blood pressure, respiratory rate and aural [tympanic] temperature) will be measured at Screening, Day -1, within 1 hour predose on dosing days, and 2 and 4 hours postdose on Day 1, Day 7 and Day 14, and at Day 15 and the EOS/Follow-up or ET visit. Vital signs will be taken after approximately 5 minutes rest in a semi-recumbent or supine position. Where assessments coincide, vital signs should be conducted first, followed by ECGs, then blood draws for PK/safety labs.

j Urine drug screen (including cotinine) and ethanol breath test to be performed.

k Includes testing for hepatitis C antibody (HCV), hepatitis B surface antigen (HbsAg), and human immunodeficiency virus (HIV) antibody. May also include testing for COVID-19 (using a locally approved test) at the Investigator’s discretion.

l For WOCBP, a serum pregnancy test is to be performed at the Screening visit, and a urine pregnancy test will be performed at all other timepoints as indicated.

m Subjects are required to fast for at least 4 hours prior to collection of blood samples for biochemistry, hematology and coagulation analysis, and a urine sample for urinalysis. Samples are to be collected prior to dosing where applicable.

^n^ Thyroid function testing, TSH, fT3, fT4 anti-TG and anti-TPO be taken at Day -1 and at 24 hours postdose (Day 2), Day 7, Day 14 and at EOS or ET.

o Administration of study drug is to occur every 12 hours (± 0.5 hours), and a final single dose is to be administered on Day 14 (morning dose only).

^p^ For Part A MAD the criteria for the fasted state on Day 1 and Day 14 of the dosing schedule will be at least 10 hours overnight prior to dosing, which is to be continued for 4 hours following dose administration. On all other days of the dosing schedule (Days 2 to 13) the criteria for the fasted state will be a modified fast of 2 hours prior to dosing and 2 hours after dosing.

q Blood samples for PK assessments will be taken at the timepoints described in Table S3. An additional blood sample for PK analysis may be collected at the EOS/Follow-up or ET visit, if appropriate.

r Pooled urine collections will be collected predose and over the intervals described in Table S3.

s Blood samples for PD assessments will be taken predose, and at 2, 8 and 12 hours postdose on Day 1, predose on Day 7, and predose and at 2, 8 and 12 hours postdose on Day 14.

Schedule of Assessments – Food Effect/Proton Pump Inhibitor Cohort

|  | Screening | Treatment Period 1 (Food Effect) a | | | | Treatment Period 2 (Food Effect) a | | | Treatment Period 3  **(PPI)** | | | | | | | EOS/ Follow-upb | ET Visitc |
| --- | --- | --- | --- | --- | --- | --- | --- | --- | --- | --- | --- | --- | --- | --- | --- | --- | --- |
| Study Day | -28 to -2 | -1 | 1 | 2 | 3 | 4 | 5 | 6 | 7 | 8-11 | 12 | 13 | 14 | 15 | 16 | Day 19 ± 1 | - |
| Study visit (CRU) | X | X | | | | X | | | X | | | | | | | X | X |
| Informed consent | X |  |  |  |  |  |  |  |  |  |  |  |  |  |  |  |  |
| Demographics | X |  |  |  |  |  |  |  |  |  |  |  |  |  |  |  |  |
| Medical history/ concurrent conditions | X |  |  |  |  |  |  |  |  |  |  |  |  |  |  |  |  |
| Smoking history | X | X |  |  |  |  |  |  |  |  |  |  |  |  |  |  |  |
| Inclusion/exclusion criteriad | X | X | X |  |  |  |  |  |  |  |  |  |  |  |  |  |  |
| Randomizatione |  | X^e^ |  |  |  |  |  |  |  |  |  |  |  |  |  |  |  |
| 12-lead ECGf | X | X | X | X |  | X | X |  |  |  | X | X | X | X |  | X | X |
| Physical examinationg | X | X |  |  |  | X |  |  |  |  |  | X |  |  |  | X | X |
| Height/weight/BMIh | X | X |  |  |  |  |  |  |  |  |  |  |  |  |  | X | X |
| Vital signsi | X | X | X | X | X | X | X | X | X | X | X | X | X | X | X | X | X |
| Drugs of abuse/alcoholj | X | X |  |  |  |  |  |  |  |  |  |  |  |  |  |  |  |
| Virologyk | X |  |  |  |  |  |  |  |  |  |  |  |  |  |  |  |  |
| Pregnancy test (WOCBP only)l | X | X |  |  |  |  |  |  |  |  |  |  |  |  |  | X | X |

Schedule of Assessments – Food Effect/Proton Pump Inhibitor Cohort (Continued)

|  | Screening | Treatment Period 1 (Food Effect)^a^ | | | | Treatment Period 2 (Food Effect)^a^ | | | Treatment Period 3  (PPI) | | | | | | | EOS/ Follow-upb | ET Visit^c^ |
| --- | --- | --- | --- | --- | --- | --- | --- | --- | --- | --- | --- | --- | --- | --- | --- | --- | --- |
| Study Day | -28 to -2 | -1 | 1 | 2 | 3 | 4 | 5 | 6 | 7 | 8-11 | 12 | **13** | **14** | **15** | 16 | Day 19 ± 1 | - |
| FSH test (postmenopausal women only) | X |  |  |  |  |  |  |  |  |  |  |  |  |  |  |  |  |
| Clinical laboratory tests (blood and urine)^m^ | X | X |  |  |  | X |  |  |  |  | X |  |  |  |  | X | X |
| Thyroid function testing^n^ |  | X |  | X |  |  | X |  |  |  |  |  | X |  |  | X | X |
| RBN-3143 administration |  |  | X |  |  | X |  |  |  |  |  | X |  |  |  |  |  |
| Pantoprazole administration |  |  |  |  |  |  |  |  | X | X | X | X |  |  |  |  |  |
| Fastingo |  | X | X |  | X | X |  |  |  |  | X | X |  |  |  |  |  |
| Standardized mealp |  |  | X |  |  | X |  |  |  |  |  |  |  |  |  |  |  |
| Blood sample for PK^q^ |  |  | X | X | X | X | X | X | X |  |  | X | X | X | X | Xr | Xr |
| Prior and concomitant medications | X | X | X | X | X | X | X | X | X | X | X | X | X | X | X | X | X |
| Adverse events | X | X | X | X | X | X | X | X | X | X | X | X | X | X | X | X | X |

Abbreviations: anti-TG = anti-thyroglobulin antibody; anti-TPO = anti-thyroid peroxidase antibody; BMI = body mass index; CRU = clinical research unit; ECG = electrocardiogram; ET = early termination; EOS = end of study; FSH = follicle stimulating hormone; fT3 = free triiodothyronine; fT4 = free thyroxine; PK = pharmacokinetics; TSH = thyroid-stimulating hormone; WOCBP = women of childbearing potential.

a Treatment Periods 1 and 2 will involve dose administration in a fed (ie, after consuming a high-fat meal) or fasted state (ie, after fasting for at least 10 hours prior to dosing) based on treatment sequence randomization.

b Subjects will be required to return to the CRU for an EOS/Follow-up visit at 5 to 7 days after the final dose of study drug (2 to 4 days after completion of Treatment Period 3).

c Subjects who withdraw early or are discontinued from the study will be encouraged to return to the CRU for an Early Termination visit (EOS assessment).

d Eligibility will be confirmed at Screening, Day -1/prior to dosing on Day 1.

e The order of treatment sequence for each subject will be determined by the results of randomization. Subjects randomized to Treatment Sequence A will receive RBN-3143 in the fasted state on Day 1 and RBN-3143 in the fed state on Day 4 and subjects randomized to Treatment Sequence B will receive RBN-3143 in the fed state on Day 1 and RBN‑3143 in the fasted state on Day 4. Randomization will take place at Day -1. Period 3 will be the same for all subjects.

f Triplicate ECGs (each reading taken at least 1 minute apart) will be taken at Screening and prior to dosing on Day 1 of whichever treatment period is first in sequence for a given subject, to establish baseline. All other ECGs (Day -1 and postdose) will be single readings unless repeat ECG is indicated. ECGs will be taken at Screening, Day -1 and within 1 hour predose, 1, 2, 4 hours, 24 hours postdose and at the EOS/post-study Follow-up or ET visit. ECGs will be taken after the subject has rested for approximately 10 minutes in a semi-recumbent or supine position. Where assessments coincide, vital signs should be conducted first, followed by ECGs, then blood draws for PK/safety labs.

g Complete physical examination required at Screening, Day -1, and predose on Day 4, Day 13, and at the EOS/Follow-up or ET visit. A symptom-directed physical examination will otherwise be performed as clinically indicated on all other days.

h At the Screening visit, height, weight and BMI measurements are required. On Day -1, weight and BMI measurements are required. At the EOS/Follow-up or ET Visit, only weight is required to be measured.

i Vital signs (pulse rate, systolic and diastolic blood pressure, respiratory rate and aural (tympanic)oral temperature) will be measured at Screening, Day -1, within 1 hour predose on Day 1 and Day 4 and Day 13, and at the following postdose timepoints: 1, 2, 3, 4, 6, 8, 10, 12 hours, 24 hours postdose and 48 hours postdose. Vital signs will also be measured on days 7 to 12, and on Day 16, and at the EOS/post-study Follow-up or ET visit. Vital signs will be assessed after the subject has been resting in a semi-recumbent or supine position for approximately 5 minutes. Where assessments coincide, vital signs should be conducted first, followed by ECGs, then blood draws for PK/safety labs.

j Urine drug screen (including cotinine) and ethanol breath test to be performed.

k Includes testing for hepatitis C antibody (HCV), hepatitis B surface antigen (HbsAg), and human immunodeficiency virus (HIV) antibody. May also include testing for COVID-19 (using a locally approved test) at the Investigator’s discretion.

^l^ For WOCBP, a serum pregnancy test is to be performed at the Screening visit, and a urine pregnancy test will be performed at all other timepoints as indicated.

^m^ Subjects are required to fast for at least 4 hours prior to collection of blood samples for biochemistry, hematology and coagulation analysis, and a urine sample for urinalysis. On Day 1, samples are to be collected prior to dosing.

^n^ Thyroid function testing, TSH, fT3, fT4 anti-TG and anti-TPO be taken at Day -1 and at 24 hours postdose (Day 2), Day 5, Day 14 and at the EOS or ET.

^o^ When RBN-3143 is administrated in a fasted state, subjects will be required to fast overnight for at least 10 hours prior to RBN-3143 administration and 4 hours following study drug administration. Water intake will be permitted as desired except for at least 1 hour before through at least 1 hour after study drug administration, except for water provided with dosing. When RBN-3143 is administered in a fed state, following an overnight fast for at least 10 hours prior to dosing, subjects will be fed a high-fat meal approximately 30 minutes prior to RBN-3143 administration.

p Based on their randomization sequence, subjects will receive RBN-3143 in either the fasted or fed state during Treatment Period 1, then in Period 2 will receive RBN-3143 in the opposite state, so that each subject is administered one fed and one fasted treatment. With the exception of the morning meal to be administered prior to fed treatment, subjects are required to fast for at least 4 hours prior to collection of blood samples for biochemistry, hematology and coagulation analysis, and a urine sample for urinalysis. Samples are to be collected prior to dosing where applicable.

^q^ Blood samples for PK assessments will be taken at the timepoints described in Table S3.

^r^ An additional blood sample for PK analysis may be collected at EOS/Follow-up or ET visit, if appropriate.

Schedule of Assessments – Drug-Drug Interaction Cohort

|  | Screening | Treatment  Period 1a | | | Treatment Period 2^a^ | | | | | | EOS/ Follow-upb | ET Visitc |
| --- | --- | --- | --- | --- | --- | --- | --- | --- | --- | --- | --- | --- |
| Study Day | -28 to -2 | -1 | 1 | 2 | 3 | 4-15 | 16 | 17 | 18 | 21 ± 1 | 24 ± 1 | - |
| Study visit (CRU) | X | X | | | X | | | | | | X | X |
| Informed consent | X |  |  |  |  |  |  |  |  |  |  |  |
| Demographics | X |  |  |  |  |  |  |  |  |  |  |  |
| Medical history/concurrent conditions | X |  |  |  |  |  |  |  |  |  |  |  |
| Smoking history | X | X |  |  |  |  |  |  |  |  |  |  |
| Inclusion/exclusion criteriad | X | X | X |  |  |  |  |  |  |  |  |  |
| 12-lead ECGe | X | X | X | X | X |  | X | X |  |  | X | X |
| Physical examinationf | X | X |  |  | X |  |  |  |  |  | X | X |
| Height/weight/BSA/BMIg | X | X |  |  |  |  |  |  |  |  | X | X |
| Vital signsh | X | X | X | X | X | X | X | X | X |  | X | X |
| Drugs of abuse/alcoholi | X | X |  |  |  |  |  |  |  |  |  |  |
| Virologyj | X |  |  |  |  |  |  |  |  |  |  |  |
| Pregnancy test (WOCBP only)k | X | X |  |  |  |  |  |  |  |  | X | X |
| FSH test (postmenopausal women only) | X |  |  |  |  |  |  |  |  |  |  |  |

Schedule of Assessments – Drug-Drug Interaction Cohort (Continued)

|  | Screening | Treatment Period 1a | | | Treatment Period 2a | | | | | | EOS/ Follow-upb | ET Visit^c^ |
| --- | --- | --- | --- | --- | --- | --- | --- | --- | --- | --- | --- | --- |
| Study Day | -28 to -2 | -1 | 1 | 2 | 3 | 4-15 | 16 | 17 | 18 | 21 ± 1 | 24 ± 1 | - |
| Clinical laboratory tests (urine)^l^ | X | X | X |  | X |  | X |  |  |  | X | X |
| Clinical laboratory tests (blood), including cystatin C^l^ | X | X | X |  | X | X | X |  | X | X | X | X |
| Thyroid function testing^m^ |  | X |  | X |  |  |  |  | X |  | X | X |
| Administration of 2 mg midazolam |  |  | X |  |  |  | X |  |  |  |  |  |
| Administration of RBN‑3143^n^ |  |  |  |  | X | X | X |  |  |  |  |  |
| Blood sample for PK^o^ |  |  | X | X | X | Xp | X | X | X |  | Xq | Xq |
| Prior and concomitant medications | X | X | X | X | X | X | X | X | X |  | X | X |
| Adverse events | X | X | X | X | X | X | X | X | X |  | X | X |

Abbreviations: anti-TG = anti-thyroglobulin antibody; anti-TPO = anti-thyroid peroxidase antibody; BMI = body mass index; BSA = body surface area; CRU = clinical research unit; ECG = electrocardiogram; EOS = end of study; ET = early termination; FSH = follicle-stimulating hormone; fT3 = free triiodothyronine; fT4 = free thyroxine; PD = pharmacodynamics; PK = pharmacokinetics; QD = once daily; TSH = thyroid-stimulating hormone; WOCBP = women of childbearing potential.

^a^ Treatment Period 1 will involve a single dose of midazolam, and Treatment Period 2 will involve 14 days of RBN-3143 to allow steady-state concentrations to be reached, then co-administration of a single dose of midazolam (2 mg) + RBN-3143. Subjects will be required to remain in the CRU from Day -1 until completion of scheduled assessments on Day 18. After Day 18, subjects can leave the CRU, but must return on Day 21 ±1 day for blood chemistries to CRU or local lab (if lab can assess cystatin C) and on Day 24 ±1 day to CRU for scheduled assessments.

b Subjects will be required to return to the CRU for an EOS/Follow-up visit at 5 to 7 days after completion of Treatment Period 2 (ie on Day 24 ±1).

c Subjects who withdraw early or are discontinued from the study will be encouraged to return to the CRU for an Early Termination visit (EOS assessment).

d Eligibility will be confirmed at Screening, Day -1/prior to dosing on Day 1.

e Triplicate ECGs (each reading taken at least 1 minute apart) will be taken at Screening and prior to dosing on Day 1, to establish baseline. All other ECGs will be single readings unless repeat ECG is indicated. ECGs will be taken after the subject has rested for approximately 10 minutes in a semi-recumbent or supine position. ECGs will be taken at Day -1, on Day 1 and Day 16 within 1 hour predose, and at 1, 2, 4, and 24 hours (Day 2 and Day 17, respectively) postdose, at Day 3 (first day of Treatment Period 2 RBN-3143 QD administration), and at the EOS/post-study Follow-up or ET visit. Where assessments coincide, vital signs should be conducted first, followed by ECGs, then blood draws for PK/safety labs.

f Complete physical examination required at Screening, Day -1, predose on Day 3, and the EOS/Follow-up or ET visit. A symptom-directed physical examination will otherwise be performed as clinically indicated on all other days.

g At the Screening visit, height, weight, and BMI measurements are required. On Day -1, weight measurements are required. At the EOS/Follow-up or ET Visit, only weight is required to be measured.

h Vital signs (pulse rate, systolic and diastolic blood pressure, respiratory rate and aural [tympanic] temperature) will be measured at Screening, Day -1, within 1 hour predose on Day 1 and days from Day 3 to Day 18, and at the following postdose timepoints on Day 1 and Day 16: 1, 2, 3, 4, 6, 8, 10, and 12 hours postdose, and 24 hours (Day 2 and Day 17, respectively), and 48 hours postdose (Day 3 and Day 18, respectively), and at the EOS/post-study Follow-up or ET visit. Vital signs will be assessed after the subject has been resting in a semi-recumbent or supine position for approximately 5 minutes. Where assessments coincide, vital signs should be conducted first, followed by ECGs, then blood draws for PK/safety labs.

i Urine drug screen (including cotinine) and ethanol breath test to be performed.

j Includes testing for hepatitis C antibody (HCV), hepatitis B surface antigen (HbsAg), and human immunodeficiency virus (HIV) antibody. May also include testing for COVID-19 (using a locally approved test) at the Investigator’s discretion.

^k^ For WOCBP, a serum pregnancy test is to be performed at the Screening visit, and a urine pregnancy test will be performed at all other timepoints as indicated.

^l^ Subjects are required to fast for at least 4 hours prior to collection of blood samples for biochemistry, hematology and coagulation analysis, and a urine sample for urinalysis. On Day 1 and Day 16, samples are to be collected prior to dosing. Blood chemistries only are performed on Days 6, 9, and 12. On Day 21, blood clinical safety labs can be performed at CRU or local lab (if lab can assess cystatin C).

^m^ Thyroid function testing, TSH, fT3, fT4 anti-TG and anti-TPO be taken at Day -1 and at 24 hours postdose (Day 2), Day 18, and at the EOS or ET.

^n^ For the DDI cohort the criteria for the fasted state on Day 1 and Day 3 and Day 16 of the dosing schedule will be at least 10 hours overnight prior to dosing, which is to be continued for 4 hours following dose administration. On all other days of the dosing schedule the criteria for the fasted state will be a modified fast of 2 hours prior to dosing and 2 hours after dosing, unless otherwise instructed.

^o^  Blood samples for PK assessments will be taken following midazolam administration at the timepoints described in Table S3.

^p^ Additional samples will be collected on Days 13, 14, and 15, 30 minutes prior to AM dosing.

^q^ An additional blood sample for PK analysis may be collected at the EOS/Follow-up or ET visit, if appropriate.

**Table S3.** Plasma and urine PK sampling schedules.

Plasma and Urine PK sampling schedule – SAD Cohorts

| Study Day | Plasma Sampling Time | Urine Sampling Time |
| --- | --- | --- |
| Day 1 | Predose (within 60 minutes prior to dosing) | Predose (within 60 minutes prior to dosing) |
|  | Postdose: 15 minutes (± 5 minutes), 30 minutes (± 5 minutes),  45 minutes (± 5 minutes),  1 hour (± 10 minutes),  1.5 hours (± 10 minutes),  2 hours (± 10 minutes),  3 hours (± 10 minutes),  4 hours (± 20 minutes),  5 hours (± 20 minutes),  8 hours (± 30 minutes),  12 hours (± 1 hour) | Postdose: Pooled collections at the following intervals (± 15 minutes): 0 to 4 hours, 4 to 8 hours, 8 to 12 hours, and 12 to 24 hours. |
| Day 2 | Postdose: 24 hours (± 1 hour), 36 hours (± 1 hour) |  |
| Day 3 | Postdose: 48 hours (± 1 hour), 60 hours (± 1 hour) | - |
| Day 4 | Postdose: 72 hours (± 1 hour) | - |
| EOS/Follow-up/ET visit | If appropriate | - |

Abbreviations: EOS = end of study; ET = early termination.

Plasma and Urine PK sampling schedule - MAD Cohorts

| Study Day | Plasma Sampling Time | Urine Sampling Time |
| --- | --- | --- |
| Day 1 and Day 14 | Predose (within 60 minutes prior to dosing) | Predose (within 60 minutes prior to dosing) |
|  | Post first dose only: 15 minutes (± 5 minutes), 30 minutes (± 5 minutes), 45 minutes (± 5 minutes), 1 hour (± 10 minutes), 1.5 hours (± 10 minutes), 2 hours (± 10 minutes), 3 hours (± 10 minutes), 4 hours (± 20 minutes), 5 hours (± 20 minutes), 8 hours (± 30 minutes), 12 hours (± 1 hour) | Post first dose only: Pooled collections at the following intervals (± 15 minutes): 0 to 4 hours,  4 to 8 hours, and 8 to 12 hours. |
| Day 2 to Day 13 (each day) | Predose: 15 minutes (± 5 minutes) | - |
| EOS/Follow-up/ET Visit | If appropriate | - |

Abbreviations: EOS = end of study; ET = early termination.

Plasma PK sampling schedule - Food Effect/PPI Cohort

| Study Day | Plasma Sampling Time |
| --- | --- |
| Day 1, Day 4**a**, Day 13 | Predose (within 60 minutes prior to dosing) |
|  | Postdose: 15 minutes (± 5 minutes), 30 minutes (± 5 minutes), 45 minutes (± 5 minutes), 1 hour (± 10 minutes), 1.5 hours (± 10 minutes), 2 hours (± 10 minutes), 3 hours (± 10 minutes), 4 hours (± 20 minutes), 5 hours (± 20 minutes),  8 hours (± 30 minutes), 12 hours (± 1 hour) |
| Day 2, Day 5, Day 14 | Postdose: 24 hours (± 1 hour), 36 hours (± 1 hour) |
| Day 3, Day 6, Day 15 | Postdose: 48 hours (± 1 hour), 60 hours (± 1 hour) |
| Day 4, Day 7, Day 16 | Postdose: 72 hours (± 1 hour) |
| EOS/Follow-up/ET Visit | If appropriate |

Abbreviations: EOS = end of study; ET = early termination.

a The Day 4 pre-dose and Day 4 post-dose 72 hour samples are 1 blood draw if dosing of second period occurs on Day 4. If Day 4 dosing is delayed for any reason, a 72-hour postdose sample should be collected and then a pre-dose sample should be collected once Treatment Period 2 begins.

Plasma PK sampling schedule – Drug-Drug Interaction Cohort

| Study Day (of each Treatment Period) | Plasma Sampling Time |
| --- | --- |
| Day 1 | Predose (within 60 minutes prior to dosing) |
|  | Postdose: 15 minutes (± 5 minutes), 30 minutes (± 5 minutes), 45 minutes (± 5 minutes), 1 hour (± 10 minutes), 1.5 hours (± 10 minutes),  2 hours (± 10 minutes), 3 hours (± 10 minutes),  4 hours (± 20 minutes), 5 hours (± 20 minutes),  8 hours (± 30 minutes), 12 hours (± 1 hour) |
| Day 2 | Postdose: 24 hours (± 1 hour), 36 hours (± 1 hour) |
| Day 3 | Predose (within 60 minutes prior to dosing) |
|  | Postdose: 15 minutes (± 5 minutes), 30 minutes (± 5 minutes), 45 minutes (± 5 minutes), 1 hour (± 10 minutes), 1.5 hours (± 10 minutes),  2 hours (± 10 minutes), 3 hours (± 10 minutes),  4 hours (± 20 minutes), 5 hours (± 20 minutes),  8 hours (± 30 minutes), 12 hours (± 1 hour) |
| Days 13, 14, 15 | 30 minutes pre-AM dose |
| Day 16 | Predose (within 60 minutes prior to dosing) |
|  | Postdose: 15 minutes (± 5 minutes), 30 minutes (± 5 minutes), 45 minutes (± 5 minutes), 1 hour (± 10 minutes), 1.5 hours (± 10 minutes),  2 hours (± 10 minutes), 3 hours (± 10 minutes),  4 hours (± 20 minutes), 5 hours (± 20 minutes),  8 hours (± 30 minutes), 12 hours (± 1 hour) |
| Day 17 | Postdose: 24 hours (± 1 hour), 36 hours (± 1 hour) |
| Day 18 | Postdose: 48 hours (± 1 hour) |
| EOS/Follow-up/ET Visit | If appropriate |

Abbreviations: EOS = end of study; ET = early termination.

**Table S4.** Demographics and baseline characteristics.

| **Variable** | **SAD**  **(n =53)** | **MAD**  **(n=24)** | **Food/PPI**  **(n=13)** | **DDI**  **(n=15)** | **All Participants**  **(N=105)** |
| --- | --- | --- | --- | --- | --- |
| **Age, years ^a^** |  |  |  |  |  |
| Mean | 26.7 | 27.8 | 30 | 30 | 27.8 |
| Range | 18-65 | 19-58 | 19-62 | 25-55 | 18-62 |
| **Sex, n (%)** |  |  |  |  |  |
| Males | 41 (77) | 20 (83) | 10 (77) | 10 (67) | 81 (77) |
| Female | 12 (33) | 4 (17) | 3 (23) | 5 (33) | 24 (23) |
| **Baseline Weight, kg** |  |  |  |  |  |
| Mean | 76.7 | 77.7 | 75.4 | 76 | 76.7 |
| Range | 50.5-104.4 | 50.5-104.4 | 57.9-93.2 | 52.5-105.8 | 50.5-105.8 |
| **Race, n (%)** |  |  |  |  |  |
| Asian | 7 (13) | 7 (29) | 1 (8) | 2 (13) | 17 (15) |
| Black | 1 (2) | 0 | 0 | 0 | 1 (1) |
| White | 37 (70) | 15 (63) | 12 (92) | 11 (73) | 75 (71) |
| Native Hawaiian/ Other Pacific Islander | 0 |  | 0 | 2 (13) | 2 (2) |
| Other | 8 (15) | 2 (8) | 0 | 0 | 10 (10) |
| **Ethnicity, n (%)** |  |  |  |  |  |
| Hispanic or Latino | 7 (13) | 2 (8) | 1 (8) | 0 | 10 (10) |
| Not Hispanic or Latino | 46 (87) | 22 (92) | 12 (92) | 15 (100) | 95 (90) |

^a^ Calculated at the date of informed consent.

Table S5: Summary of Treatment-Related TEAEs – Single Ascending Doses.

|  | RBN-3143 25 mg  N=5  n (%) | RBN-3143 50 mg  N=6  n (%) | RBN-3143 100 mg  N=6  n (%) | RBN-3143 150 mg  N=6  n (%) | RBN-3143  300 mg  N=5  n (%) | RBN-3143  600 mg  N=6  n (%) | RBN-3143  1000 mg  N=6  n (%) | Overall  RBN-3143  N=40  n (%) | Placebo  N=13  n (%) |
| --- | --- | --- | --- | --- | --- | --- | --- | --- | --- |
| **Subjects with at least one treatment-related TEAE** | 0 | 3 (50.0) | 2 (33.3) | 1 (16.7) | 1 (20.0) | 1 (16.7) | 0 | 8 (20.0) | 1 (7.7) |
| **Ear pruritus** | 0 | 0 | 0 | 0 | 0 | 1 (16.7) | 0 | 1 (2.5) | 0 |
| **Vision blurred** | 0 | 0 | 0 | 0 | 0 | 0 | 0 | 0 | 1 (7.7) |
| **Nausea** | 0 | 1 (16.7) | 0 | 0 | 1 (20.0) | 0 | 0 | 2 (5.0) | 0 |
| **Vomiting** | 0 | 1 (16.7) | 0 | 0 | 1 (20.0) | 0 | 0 | 2 (5.0) | 0 |
| **Blood TSH increased** | 0 | 1 (16.7) | 0 | 0 | 0 | 0 | 0 | 1 (2.5) | 1 (7.7) |
| **Headache** | 0 | 1 (16.7) | 2 (33.3) | 1 (16.7) | 0 | 0 | 0 | 4 (10.0) | 0 |
| **Presyncope** | 0 | 1 (16.7) | 0 | 0 | 0 | 0 | 0 | 1 (2.5) | 0 |
| **Somnolence** | 0 | 0 | 0 | 0 | 1 (20.0) | 0 | 0 | 1 (2.5) | 0 |
| **Throat irritation** | 0 | 0 | 0 | 0 | 0 | 1 (16.7) | 0 | 1 (2.5) | 0 |
| **Hyperhidrosis** | 0 | 0 | 0 | 0 | 0 | 0 | 0 | 0 | 1 (7.7) |

TEAEs are defined as AEs that started from the first administration of study drug until the Follow-up/EOS visit or up to a 30-day Follow-up Period.

If a subject had multiple occurrences of a TEAE, the subject is presented only once in the Subject count (n) column for a given category.

Percentages are calculated (the denominator used for the calculation) based on the number of subjects in the safety population in each treatment group (N).

Table S6: Summary of Treatment-Related TEAEs – Multiple Ascending Doses.

|  | RBN-3143  150 mg  N=6  n (%) | RBN-3143  300 mg  N=6  n (%) | RBN-3143  500 mg  N=6  n (%) | Overall  RBN-3143  N=18  n (%) | Placebo  N=6  n (%) |
| --- | --- | --- | --- | --- | --- |
| **Subjects with at Least One Treatment-Related TEAE** | 1 (16.7%) | 0 | 3 (50.0%) | 4 (22.2%) | 1 (16.7%) |
| **Nausea** | 0 | 0 | 1 (16.7%) | 1 (5.6%) | 0 |
| **Paresthesia oral** | 0 | 0 | 0 | 0 | 1 (16.7%) |
| **Vomiting** | 0 | 0 | 0 | 0 | 1 (16.7%) |
| **Vascular access site pain** | 0 | 0 | 1 (16.7%) | 1 (5.6%) | 0 |
| **Blood TSH increased** | 0 | 0 | 2 (33.3%) | 2 (11.1%) | 1 (16.7%) |
| **Serum creatinine increased** | 0 | 1 (16.7%) | 0 | 1 (16.7%) | 0 |
| **Headache** | 0 | 0 | 1 (16.7%) | 1 (5.6%) | 0 |
| **Paresthesia** | 0 | 0 | 0 | 0 | 1 (16.7%) |
| **Dry skin** | 1 (16.7%) | 0 | 0 | 1 (5.6%) | 0 |
| **Pruritus** | 1 (16.7%) | 0 | 0 | 1 (5.6%) | 0 |

TEAEs are defined as AEs that started from the first administration of study drug until the Follow-up/EOS visit or up to a 30-day Follow-up Period.

If a subject had multiple occurrences of a TEAE, the subject is presented only once in the Subject count (n) column for a given category.

Percentages are calculated (the denominator used for the calculation) based on the number of subjects in the safety population in each treatment group (N).
